# Supplementary material for: A socioeconomic and cost benefit analysis of Tropical Race 4 (TR4) prevention methods among banana producers in Colombia
Source: PLoS One. 2024 Oct 30;19(10):e0311243. doi: 10.1371/journal.pone.0311243 (PMC11524470; doi:10.1371/journal.pone.0311243)
Supplement: S1 Table — (DOCX) [file pone.0311243.s001.docx]

**Supporting information**

**Table A. Mean t-tests for comparing averages between departments.**

| **Variable** | **Stat** | **Antioquia** | **Magdalena** | **Difference** |
| --- | --- | --- | --- | --- |
| Farm area (ha) | Mean | 134.56 | 12.19 | 122.37*** |
|  | Std. err | (49.71) | (32.04) | - |
|  | N | 14 | 175 | - |
| Plant seedlings (B)  (Yes = 1, No = 0) | Mean | 0.07 | 0.36 | -0.29** |
|  | Std. err | 0.07 | 0.04 | - |
|  | N | 15 | 176 | - |
| Plant corms (B)  (Yes = 1, No = 0) | Mean | 0.93 | 0.64 | 0.29** |
|  | Std. err | 0.07 | 0.04 | - |
|  | N | 15 | 176 | - |
| Yield (tons/ha/year) | Mean | 46.24 | 38.66 | 7.58 |
|  | Std. err | 4.12 | 1.2 |  |
|  | N | 11 | 160 |  |
| Heard about TR4 (B)  (Yes = 1, No = 0) | Mean | 1 | 0.99 | 0.01 |
|  | Std. err | (0) | (0.01) | - |
|  | N | 15 | 176 | - |
| Number of TR4 trainings received | Mean | 8.07 | 5.81 | 2.26 |
|  | Std. err | 1.22 | 0.48 | - |
|  | N | 14 | 159 | - |
| Implemented a monitoring strategy? (B) (Yes = 1, No = 0) | Mean | 1 | 0.9 | 0.1 |
|  | Std. err | 0 | 0.02 | - |
|  | N | 15 | 175 | - |
| Built disinfection station (B) (Yes = 1, No = 0) | Mean | 1 | 0.78 | 0.22** |
|  | Std. err | 0 | 0.03 | - |
|  | N | 15 | 174 | - |
| Producer disinfects shoes (B) (Yes = 1, No = 0) | Mean | 0.73 | 0.7 | 0.03 |
|  | Std. err | 0.46 | 0.5 |  |
|  | N | 15 | 174 |  |
| Producer disinfects tool/machinery (B) (Yes = 1, No = 0) | Mean | 0.2 | 0.21 | -0.01 |
|  | Std. err | 0.11 | 0.03 | - |
|  | N | 15 | 174 | - |
| Built cement paths (B)  (Yes = 1, No = 0) | Mean | 0.33 | 0.05 | 0.28*** |
|  | Std. err | 0.13 | 0.02 | - |
|  | N | 15 | 174 | - |

Notes: (B): binary variables tested using the Fisher’s exact test and (N) is number. Level of significance is: *p < 0.10, **p < 0.05, and ***p < 0.01.
