# Supplementary material for: A socioeconomic and cost benefit analysis of Tropical Race 4 (TR4) prevention methods among banana producers in Colombia
Source: PLoS One. 2024 Oct 30;19(10):e0311243. doi: 10.1371/journal.pone.0311243 (PMC11524470; doi:10.1371/journal.pone.0311243)
Supplement: Attachment — Submitted filename: PONE-D-24-08984_reviewer. RevGR.pdf [file pone.0311243.s003.pdf]

PLOS ONE

A socioeconomic and cost benefit analysis of Tropical Race 4 (TR4) among producers in Colombia

--Manuscript Draft--

**Manuscript Number:**	PONE-D-24-08984
**Article Type:**	Research Article
**Full Title:**	A socioeconomic and cost benefit analysis of Tropical Race 4 (TR4) among producers in Colombia
**Short Title:**	Fusarium Tropical Race 4: A socioeconomic and cost benefit analysis
**Corresponding Author:**	Thea Ritter<br>Alliance of Bioversity International and CIAT: Alliance of Bioversity International and International Center for Tropical Agriculture<br>Cali, COLOMBIA
**Keywords:**	Fusarium Tropical Race 4; Fusarium wilt; cost benefit analysis; Banana; socioeconomic analysis, Colombia.
**Abstract:**	The global banana industry faces a significant threat from Fusarium oxysporum f. sp. cubense Tropical Race 4 (TR4). While prior research has concentrated on TR4's dissemination, reproductive conditions, and resistant banana varieties, this study employs a socioeconomic and cost-benefit analysis to explore the susceptibility of banana producers to TR4. It assesses the financial viability of current monitoring strategies and estimates potential losses in the event of TR4 spreading within the study area. Interviews were conducted with producers and key stakeholders in Colombia's top two banana-producing departments, Antioquia and Magdalena. The findings reveal that farming systems are highly vulnerable to TR4, particularly due to the prevalent use of corms. Producers employ preventive measures such as cement paths, fences, disinfectant stations, and footbaths to counteract TR4's spread. A cost-benefit analysis indicates that the benefits of these mitigation methods significantly outweigh the associated costs. Considering the substantial impact TR4 could have in Colombia if it becomes more widespread, we recommend widespread adoption of preventive measures, including the construction and utilization of cement paths and disinfectant methods on all banana farms. Additionally, to enhance awareness and early detection, we propose leveraging technology, such as mobile applications (apps) and chat groups, to empower farmers in identifying and preventing the spread of TR4.
**Order of Authors:**	Thea Ritter
	Diego Álvarez
	Leslie Estefany Mosquera
	Jonathan Mockshell
	Edward Martey
**Additional Information:**	
**Question**	**Response**
**Financial Disclosure**<br><br>Enter a financial disclosure statement that describes the sources of funding for the work included in this submission. Review the submission guidelines for detailed requirements. View published research articles from *PLOS ONE* for specific examples.	Yes

Powered by Editorial Manager® and ProduXion Manager® from Aries Systems Corporation

This statement is required for submission and **will appear in the published article** if the submission is accepted. Please make sure it is accurate.<br><br>**Funded studies**<br>Enter a statement with the following details:<br>• Initials of the authors who received each award<br>• Grant numbers awarded to each author<br>• The full name of each funder<br>• URL of each funder website<br>• Did the sponsors or funders play any role in the study design, data collection and analysis, decision to publish, or preparation of the manuscript?<br><br>Did you receive funding for this work?	
Please add funding details.<br>as follow-up to "**Financial Disclosure**<br><br>Enter a financial disclosure statement that describes the sources of funding for the work included in this submission. Review the submission guidelines for detailed requirements. View published research articles from *PLOS ONE* for specific examples.<br><br>This statement is required for submission and **will appear in the published article** if the submission is accepted. Please make sure it is accurate.<br><br>**Funded studies**<br>Enter a statement with the following details:<br>• Initials of the authors who received each award<br>• Grant numbers awarded to each author<br>• The full name of each funder<br>• URL of each funder website<br>• Did the sponsors or funders play any role in the study design, data collection and analysis, decision to publish, or preparation of the manuscript?	This work was funded by the Deutsche Gesellschaft für Internationale Zusammenarbeit (GIZ) in the project, "Innovations for the prevention and management of the banana fungal disease Foc TR4 (ALER4TA)", implemented by the Fund for the Promotion in Agriculture (i4Ag) as part of the special initiative Transformation of Agricultural and Food Systems.

Powered by Editorial Manager® and ProduXion Manager® from Aries Systems Corporation

Did you receive funding for this work?"	
Please select the country of your main research funder (please select carefully as in some cases this is used in fee calculation).<br>as follow-up to "**Financial Disclosure**<br><br>Enter a financial disclosure statement that describes the sources of funding for the work included in this submission. Review the submission guidelines for detailed requirements. View published research articles from *PLOS ONE* for specific examples.<br><br>This statement is required for submission and **will appear in the published article** if the submission is accepted. Please make sure it is accurate.<br><br>**Funded studies**<br>Enter a statement with the following details:<br>• Initials of the authors who received each award<br>• Grant numbers awarded to each author<br>• The full name of each funder<br>• URL of each funder website<br>• Did the sponsors or funders play any role in the study design, data collection and analysis, decision to publish, or preparation of the manuscript?<br><br>Did you receive funding for this work?"	GERMANY - DE
**Competing Interests**<br><br>Use the instructions below to enter a competing interest statement for this submission. On behalf of all authors, disclose any competing interests that could be perceived to bias this work—acknowledging all financial support and any other relevant financial or non-financial competing interests.<br><br>This statement is required for submission and **will appear in the published article** if	The authors have declared that no competing interests exist.

the submission is accepted. Please make sure it is accurate and that any funding sources listed in your Funding Information later in the submission form are also declared in your Financial Disclosure statement.<br><br>View published research articles from *PLOS ONE* for specific examples.<br><br>**NO authors have competing interests**<br><br>Enter: *The authors have declared that no competing interests exist.*<br><br>**Authors with competing interests**<br><br>Enter competing interest details beginning with this statement:<br><br>*I have read the journal's policy and the authors of this manuscript have the following competing interests: [insert competing interests here]*<br><br>* typeset	
**Ethics Statement**<br><br>Enter an ethics statement for this submission. This statement is required if the study involved:<br><br>• Human participants<br>• Human specimens or tissue<br>• Vertebrate animals or cephalopods<br>• Vertebrate embryos or tissues<br>• Field research<br><br>Write "N/A" if the submission does not require an ethics statement.<br><br>General guidance is provided below. Consult the submission guidelines for detailed instructions. **Make sure that all information entered here is included in the Methods section of the manuscript.**	Ethics approval was obtained for this study from our organization's ethics committee. The Alliance-Institutional Review Board (Alliance- IRB) reviewed the study protocol and approved this research (2022-IRB43).<br><br>Before beginning the interviews, respondents provided verbal consent for their participation in the study. They were informed that they could withdraw their consent and discontinue participation in the study at any time during the interviews. Enumerators recorded each respondent's verbal consent on the survey form.

**Format for specific study types**<br><br>**Human Subject Research (involving human participants and/or tissue)**<br>• Give the name of the institutional review board or ethics committee that approved the study<br>• Include the approval number and/or a statement indicating approval of this research<br>• Indicate the form of consent obtained (written/oral) or the reason that consent was not obtained (e.g. the data were analyzed anonymously)<br><br>**Animal Research (involving vertebrate animals, embryos or tissues)**<br>• Provide the name of the Institutional Animal Care and Use Committee (IACUC) or other relevant ethics board that reviewed the study protocol, and indicate whether they approved this research or granted a formal waiver of ethical approval<br>• Include an approval number if one was obtained<br>• If the study involved *non-human primates*, add *additional details* about animal welfare and steps taken to ameliorate suffering<br>• If anesthesia, euthanasia, or any kind of animal sacrifice is part of the study, include briefly which substances and/or methods were applied<br><br>**Field Research**<br><br>Include the following details if this study involves the collection of plant, animal, or other materials from a natural setting:<br>• Field permit number<br>• Name of the institution or relevant body that granted permission	
**Data Availability**<br><br>Authors are required to make all data underlying the findings described fully available, without restriction, and from the time of publication. PLOS allows rare exceptions to address legal and ethical concerns. See the PLOS Data Policy and FAQ for detailed information.	No - some restrictions will apply

A Data Availability Statement describing where the data can be found is required at submission. Your answers to this question constitute the Data Availability Statement and **will be published in the article**, if accepted.<br><br>**Important:** Stating 'data available on request from the author' is not sufficient. If your data are only available upon request, select 'No' for the first question and explain your exceptional situation in the text box.<br><br>Do the authors confirm that all data underlying the findings described in their manuscript are fully available without restriction?	
**Describe where the data may be found in full sentences. If you are copying our sample text, replace any instances of XXX with the appropriate details.**<br><br>• If the data are **held or will be held in a public repository**, include URLs, accession numbers or DOIs. If this information will only be available after acceptance, indicate this by ticking the box below. For example: *All XXX files are available from the XXX database (accession number(s) XXX, XXX.*).<br>• If the data are all contained **within the manuscript and/or Supporting Information files**, enter the following: *All relevant data are within the manuscript and its Supporting Information files.*<br>• If neither of these applies but you are able to provide **details of access elsewhere**, with or without limitations, please do so. For example:<br><br>*Data cannot be shared publicly because of [XXX]. Data are available from the XXX Institutional Data Access / Ethics Committee (contact via XXX) for researchers who meet the criteria for access to confidential data.*<br><br>*The data underlying the results presented in the study are available from (include the name of the third party*	Not all data can be shared publicly because of concerns of confidentiality. Some data are available from the authors (contact via Jonathan Mockshell at j.mockshell@cgiar.org) for researchers who meet the criteria for access to the data.

and contact information or URL).<br>• This text is appropriate if the data are owned by a third party and authors do not have permission to share the data.<br><br>* typeset	
Additional data availability information:	

Manuscript
Click here to access/download;Manuscript;Manuscript_revision 03.08.2024.docx

A socioeconomic and cost benefit analysis of Tropical Race 4 (TR4) among banana producers in Colombia

Thea Ritter[1]

(t.ritter@cgiar.org)

Diego Álvarez[1]

(D.A.Alvarez@cgiar.org)

Leslie Estefany Mosquera[1]

(L.Mosquera@cgiar.org)

Jonathan Mockshell[1]

(j.mockshell@cgiar.org)

Edward Martey [1,2]

(eddiemartey@gmail.com)

[1]Performance, Innovation and Strategic Analysis for Impact (PISA4Impact), Applied Economics and Impact Evaluation, International Center for Tropical Agriculture (CIAT)

[2]Socioeconomics section, CSIR-Savanna Agricultural Research Institute

Corresponding authors: Thea Ritter (t.ritter@cgiar.org) and Jonathan Mockshell (j.mockshell@cgiar.org)

Funding

This work was funded by the Deutsche Gesellschaft für Internationale Zusammenarbeit (GIZ) in the project, "Innovations for the prevention and management of the banana fungal disease Foc TR4 (ALER4TA)", implemented by the Fund for the Promotion in Agriculture (i4Ag) as part of the special initiative Transformation of Agricultural and Food Systems.

Acknowledgments

Special thanks to Jorge Eliecer Vargas who provided valuable guidance and information during the socioeconomic study. We are grateful for the funding for this work from the Deutsche Gesellschaft für Internationale Zusammenarbeit (GIZ). We are thankful for the support in the data collection process to the banana producer associations Augura and Asbama, the producer cooperatives, members from the Latin American and Caribbean Network of Fair-Trade Small Producers and Workers (CLAC, for its acronym in Spanish) and, last but not least, to Leonardo García and his data collection team. The opinions expressed are those of the authors, and do not necessarily reflect those of the funders or institutions of affiliation.

Fusarium Tropical Race 4 (TR4) in Colombia: A dire situation for banana producers and recommendations for combating the disease

Abstract

The global banana industry faces a significant threat from *Fusarium oxysporum* f. sp. *cubense* Tropical Race 4 (TR4). While prior research has concentrated on TR4's dissemination, reproductive conditions, and resistant banana varieties, this study employs a socioeconomic and cost-benefit analysis to explore the susceptibility of banana producers to TR4. It assesses the financial viability of current monitoring strategies and estimates potential losses in the event of TR4 spreading within the study area. Interviews were conducted with producers and key stakeholders in Colombia's top two banana-producing departments, Antioquia and Magdalena. The findings reveal that farming systems are highly vulnerable to TR4, particularly due to the prevalent use of corms. Producers employ preventive measures such as cement paths, fences, disinfectant stations, and footbaths to counteract TR4's spread. A cost-benefit analysis indicates that the benefits of these mitigation methods significantly outweigh the associated costs. Considering the substantial impact TR4 could have in Colombia if it becomes more widespread, we recommend widespread adoption of preventive measures, including the construction and utilization of cement paths and disinfectant methods on all banana farms. Additionally, to enhance awareness and early detection, we propose leveraging technology, such as mobile applications (apps) and chat groups, to empower farmers in identifying and preventing the spread of TR4.

Keywords: Fusarium Tropical Race 4; Fusarium wilt; cost benefit analysis; banana; socioeconomic analysis, Colombia.

1. Introduction

The global banana industry faces a significant threat from *Fusarium oxysporum* f. sp. *cubense* (Foc) Tropical Race 4 (TR4), a soil-borne fungus that targets the vascular system of banana plants, impeding water and nutrient transport and ultimately leading to plant death. TR4 poses a persistent threat as it can remain in the soil for over 50 years (Pegg et al., 2019), causing widespread destruction in affected areas. This fungus not only inflicts substantial economic losses on banana crops but also raises concerns for various other crops (Okungbowa and Shittu, 2013). Foc exhibits diverse populations, with TR4 being the sole strain capable of affecting the economically vital Cavendish cultivar. TR4 also impacts a wide array of local cultivars that remain unaffected by Foc Race 1 (R1) and Race 2 (R2). Remarkably, over 80% of the global banana and plantain production relies on germplasms susceptible to TR4 (Staver et al., 2020). Recognized as the most aggressive Foc strain, TR4 poses a formidable threat to banana cultivation worldwide (Buddenhagen, 2009).

The emergence of TR4 poses a significant threat to global banana production, as it lacks an effective treatment, leading to complete yield loss. There are no commercially available varieties resistant to this persistent disease. TR4 was initially identified in Asia in the 1970s, later spreading to Africa in 2013 and reaching Latin America with detections in Colombia in 2019, Peru in 2021, and Venezuela in 2023. Arrival of TR4 in Colombia put the region's banana industry and greater economy at risk. Four of the world's five leading banana exporters are in Latin America: Ecuador, Costa Rica, Guatemala, and Colombia (FAOSTAT, 2022). In Colombia, banana exports were valued at $916.2 million USD in 2020, representing 11.6% of total agricultural exports (Augura, 2021). Beyond its impact on international trade, the banana sector in Colombia holds significance for food security and employment, providing 293,648 direct and indirect jobs in the country (MADR, 2022). To address the threat of TR4 on banana production in Colombia, three key interventions have been implemented: enhancing the

capacity of stakeholders to manage TR4, reinforcing the use of preventive measures through surveillance, and containing existing cases of TR4.

Despite the importance of bananas in Colombia and the recent spread of TR4 there, there are no studies on the social and economic consequences of TR4 in Latin America, or on the effectiveness of interventions in Colombia or any other Latin American country, and no studies on the socioeconomic effects of TR4 on banana value chain actors. Important aspects such as the disease's effects on food security and the livelihoods of value chain actors are absent. Instead, the scant literature focuses on suitable environmental conditions for TR4 and its geographical spread (Barahona, 2021; Cook et al., 2012, 2015; Olivares et al., 2021; Pemsl and Staver, 2014; Staver et al., 2020), the efficacy of control methods (Damodaran et al., 2019; Limbaga et al., 2023), and overall costs of TR4 at the farm-level (Aquino et al., 2013) and country-level in Asia (Malik et al., 2013; Molano and Montoya, 2022). A few studies focus on evaluating the impact of strategies to mitigate the effects of TR4 and other banana diseases on poverty reduction and avoided production losses. These studies use methodologies such as cost benefit analysis (CBA), net present value (NPV), internal rate of return (IRR), and partial equilibrium economic surplus models to compare strategies under various assumptions related to the adoption of different scenarios and spread rates (Hort Innovation, 2020; Scheerer et al., 2018a, 2018b; Staver et al., 2020). For instance, Scheerer et al. (2018a) evaluate the economic viability of implementing strategies to mitigate the impact of Fusarium (R1, R2, STR4, and TR4) in Asia, Africa, and Latin America, utilizing CBA to calculate NPV and IRR. Results indicate that, assuming a 50% spread rate, production losses over a 25-year horizon could reach nearly 1.7 million hectares in the 29 countries analyzed. (Scheerer et al., 2018a). Staver et al. (2020) examine the impact of four mitigation options against TR4. The study assumes two adoption scenarios, low and high adoption, using a partial equilibrium economic surplus model and CBA, finding that all options generate positive NPV and that the IRR is above the standard

10% rate. The above studies show the immense potential impacts TR4 and the benefits of taking measures to detect and prevent its spread.

This study aims to fill the existing gaps in the literature by evaluating the socioeconomic effects of TR4 and the financial viability of mitigation strategies implemented to counteract TR4 in Colombia based on data collected from banana producers on their farming systems, food security, and TR4 mitigation measures. Through a CBA and socioeconomic analysis, this study aims to answer the following research questions: (1) How vulnerable are banana producers and current banana farming systems to TR4? (2) What monitoring strategies are currently implemented on banana farms? (3) What is the financial viability of implementing biosecurity strategies on banana farms to prevent the spread of TR4 in terms of their net present value (NPV) and benefit-cost ratio (BCR)? (4) What are the estimated economic losses if TR4 were to spread? The findings of the study will provide actionable insights into the potential of technology, policies, and institutional responses to preventing and managing the spread of TR4, thereby helping policymakers, scientists, development organizations, and other stakeholders develop and target policies and initiatives to help combat the spread of TR4.

2. Materials and methods

This section first describes the study area before detailing the data collection tools and analysis strategy.

2.1 Study area

Figure 1 shows the study area in Colombia. The selection of the departments of Antioquia and Magdalena for this study is underpinned by several strategic considerations. these departments stand out as the primary hubs for banana production in Colombia. Second, they exhibit distinct farming systems: Antioquia relies on large plantations with hired labor, while Magdalena predominantly features smaller farms relying on family labor. This deliberate choice enables a

comparative analysis of the effects of TR4 and disease management practices across different production systems. Third, Antioquia and Magdalena produce bananas for different markets: Antioquia is the only department in Colombia that produces for both export and domestic markets, whereas Magdalena (along with La Guajira) produces only for international markets. The two major varieties sold in Colombia are bananas for export (Cavendish) and bananas for domestic markets (Banano Criollo). Lastly, the inclusion of Magdalena is motivated by the presence of three confirmed TR4 cases (Iguarán, 2022), providing an opportunity to investigate areas both with and without TR4.

Figure 1. Map of departments of study in Colombia

(Insert Figure 1 here)

Source: own elaboration based on survey

2.2 Data collection and sources

Prior to initiating data collection, a workshop under the ALER4TA project was conducted in Santa Marta, Magdalena, from Ocotber 24th–25th, 2022 to ensure stakeholders were well-informed about the project's objectives and the nature of the data to be gathered. The workshop also served to identify existing TR4 mitigation actions undertaken by industry participants in Colombia, offering valuable insights that shaped the subsequent data collection process.

Both quantitative and qualitative data were gathered through a comprehensive approach involving a producer survey and semi-structured interviews (SSIs). Ethics approval was obtained for this study from our organization’s ethics committee. Before beginning the interviews, respondents provided verbal consent for their participation in the study. They were informed that they could withdraw their consent and discontinue participation in the study at any time during the interviews. Enumerators recorded each respondent’s verbal consent on the survey form.

The producer survey included 191 smallholder farmers and large plantation owners/managers (see Table 1). The number of surveys conducted in each municipality varies widely given the different production systems: A higher number of interviews were conducted in Magdalena (176), predominantly due to the prevalence of smaller-sized farms in comparison to Antioquia (15). The SSIs provided valuable insights into the diverse effects of TR4 that may not have been captured by quantitative data alone. In order to understand the varied effects of TR4 throughout the value chain, we conducted 28 SSIs with a range of stakeholders, including extension agents, banana laborers, researchers, representatives from producer organizations, and government officials (Table 1). Interviews were conducted between February 21st–July 6th, 2023. Data collection took longer than planned to complete because of having to coordinate with the schedules of associations and cooperatives to interview members and difficulty interviewing respondents with TR4, explained below.

Table 1: Sampled actors interviewed

Type of actor	Number of interviews
Producers	191
Extension agents, service providers, and multiplicators	9
Banana laborers	10
Researchers	2
Presidents/Vice Presidents of producer organizations	5
Government officials	2
Observation	219

Source: Own elaboration based on semi-structured interviews.

Despite the confirmation of TR4 on large farms in La Guajira and Magdalena, attempts to interview producers or managers from these farms were unsuccessful. Despite assuring anonymity and employing various outreach methods over several months, those who had

encountered TR4 were reluctant to participate or provide information due to concerns about potential harm to their economic activities. Producers expressed fear that revealing their TR4 situation might hinder their chances of obtaining necessary farming loans from banks. Consequently, only producers without TR4 agreed to participate in the study. Nevertheless, these participants had experience with training and managing TR4. The survey collected data on various aspects, including income from banana production, income from banana labor, the banana production system, experiences with TR4, membership and participation in producer organizations, banana consumption at home, food security, the use of technology for TR4 prevention and management, and knowledge related to TR4 detection and management.

In addition to primary data, we also utilized secondary data to estimate costs and to simulate the annual economic losses due to TR4 across four dispersion scenarios. Average operational costs per hectare for banana cultivation in each department were estimated based on publicly available data (MADR, 2022). Unfortunately, we were unable to obtain cost information from the producers themselves because they were unwilling to share this information. The simulation utilized data on hectares planted with bananas and departmental production for the year 2021 sourced from the Municipal Agricultural Assessments conducted by the Colombian Ministry of Agriculture and the average sales price from surveyed producers. The estimations of the losses caused by TR4 dispersion scenarios utilize data on hectares planted with bananas and production in 2021 from the Municipal Agricultural Assessments conducted by the Colombian Ministry of Agriculture. This data was combined with data on average sales prices from surveyed producers.

2.3 Data analysis process

Data from the producer survey and SSIs were analyzed separately to examine the far-reaching effects of TR4 on various indicators, such as food security, income, and the detection and

management of TR4. From the SSIs, open-ended questions and multiple-choice questions were tabulated in Excel.

CBA is an analytical tool that balances the costs and benefits of a project to evaluate its financial profitability (Castro et al., 2008). This study uses a CBA approach to evaluate the potential benefits of TR4 mitigation practices. Given that we had no access to cost data for all surveyed producers, a CBA was performed using average values per department rather than estimating NPVs and BCRs for each producer.

There are several factors necessary for the CBA calculation. The first is the analysis horizon, which is the time horizon or useful life of the project. Considering the Colombian National Tax Statute Article 137, we selected a 40-year analysis horizon as the foundational useful life for the disinfection stations and path cement. An annual depreciation rate of 2.22% and 2.5% was applied to disinfection stations and cement paths, respectively. The second factor is the discount rate, employed to discount future payments to their present value, thereby accounting for the time value of money. The higher the discount rate, the lower the present value of the payments. In this case, a 12% social discount rate was applied to bring future costs and benefits to their present value (Piraquive et al., 2018). This rate reflects society's time preference and allows for the comparison of cost and benefit streams on an equivalent basis in the present. The third factor is the investment cost, which is the initial cost of an implemented mitigation strategy. The last factor consists of operation and maintenance costs, which are all the costs necessary for the development and implementation of mitigation strategies, including fixed costs, input costs, repairs, and maintenance.

The steps following steps were undertaken in the CBA:

Step 1: Identify the specific activities of each of the TR4 interventions or mitigation strategies through a workshop with experts and key stakeholders.

Step 2: Collect data on TR4 mitigation strategies applied in the field and their cost structure.

Step 3: Clean and analyze the data to ensure uniform units for cost and income information, specifically on a per-hectare basis. This standardization facilitates a clearer assessment of the financial viability at the level of individual plots.

Step 4: Identify the benefits linked to TR4 mitigation strategies, considering benefits as the monetary losses averted. This entails estimating the losses that would have occurred on the farm if the banana plants had contracted TR4. Following the approach used in Cook et al. (2012), these averted losses are termed exclusion benefits.

Step 5: Calculate net cash flows by subtracting total income from total costs.

Step 6: Calculate NPV, which is a financial metric that is the difference between the present value of cash inflows and outflows associated with an investment or project. NPV helps assess the profitability or viability of an investment by determining whether it generates more value than it costs when considering the time value of money. A positive NPV indicates that the investment is likely to be financially worthwhile, while a negative NPV suggests otherwise. To calculate NPV, one needs to consider the time horizon, net cash flows, and the discount rate, all of which are incorporated into the following NPV formula:

$$NPV = \sum_{t=0}^{n} \frac{Net\ cash\ flow_t}{(1+\imath)^t} \qquad (1)$$

where n is the total number of time periods, i is the discount rate, and t is the time period.

Step 7: Adjust the income (benefit) and cost of each period to the present using the discount rate selected in the following formula:

$$Current\ benefit = \frac{Income_t}{(1+\imath)^t} \qquad (2)$$

$$Current\ cost = \frac{cost_t}{(1 + \imath)^t} \qquad (3)$$

Step 8: Estimate the BCR. As shown in the equation below, the BCR is obtained by dividing the sum of current benefits by the sum of current costs. A ratio greater than one indicates that the project will generate more benefits than costs, therefore making it financially profitable.

$$BCR = \sum_{t=0}^{n} \frac{Current\ Benefit_t}{Current\ cost_t} \qquad (4)$$

By utilizing key financial metrics, including BCR and NPV, the analysis aims to offer a thorough insight into the economic feasibility of TR4 mitigation strategies implemented by banana producers. Additionally, economic losses associated with TR4 are estimated across four dispersion scenarios (25%, 50%, 75%, and 100%), guided by the work of Scheerer et al. (2018a, 2018b).

3. Results

3.1 Background information on surveyed producers and farming systems

This section provides information on the surveyed producers and farming systems.

3.1.1 Gender, education, and primary occupation of producers

Gaining insights into the demographic characteristics of respondents is crucial for understanding the diversity of experiences and perspectives. A minority of respondents (16.2%) are women, with a slightly higher proportion in Antioquia (20%) compared to Magdalena (15.9%). All respondents in Magdalena indicated that they are able to read and write, while in Antioquia, 3.4% are unable to do so. Educational attainment in Magdalena is diverse: approximately 35.1% have a high school education, 19.3% completed technical studies, 18.1% pursued university studies, and 5.1% had not completed any formal education. In Antioquia, the majority of producers (80%) have pursued university studies, 13% completed

technical studies, and 7% finished high school. These results suggest that, on average, banana producers in Antioquia have higher education levels than those in Magdalena. Regarding primary occupation, most producers in Magdalena work on their own farm (73.1%), while 11.4% manage other farms. In Antioquia, 46.7% of respondents work on their own farm as their primary occupation, 13.3% work as managers on other banana farms, and 40% are involved in various other farming activities, such as managerial, agronomist, and administrative roles.

3.1.2 Food security

Food security was assessed using the Food Insecurity Experience Score (FIES) (FAO, n.d.), which is based on eight questions about respondents' experiences over the last 12 months, such as skipping meals or being unable to eat despite being hungry. The FIES categorizes responses into levels of food insecurity, including mild, moderate, and severe. The results indicate that producers in Antioquia have adequate access to food, as all surveyed producers did not face food insecurity. However, in Magdalena, the scenario is different, with 20.45% of producers experiencing mild food insecurity, 18.75% facing moderate insecurity, and 22.73% suffering from severe food insecurity. Figure 2 shows the questions and responses of banana producers from Magdalena related to food security.

Figure 2. Food security questions and responses from banana producers in Magdalena (N = 176).

(Insert Figure 2 here)

Source: Own elaboration based on the Food Insecurity Experience Score in the producer survey. Antioquia is not shown since all surveyed producers responded "no” to all questions there.

3.1.3 Farm size and systems

Farms are significantly larger in Antioquia than in Magdalena. The difference in the average farm size between Antioquia (134 ha) and Magdalena (12 ha) is statistically significant at the 1% level (see Table A1 in the Appendix). In Magdalena, 59% of respondents are small-scale producers (farms less than 3 ha), 29% medium-scale (farms 3 to 20 ha), and 12% large-scale (farms greater than 20 ha). All 15 respondents in Antioquia are large-scale producers. Conventional production systems dominate, with only 4% employing organic production methods. Marketing companies play an important role in pest and disease control, as they carry out aerial spraying against Black Sigatoka. In Magdalena, for example, there are signs on some banana plantations warning people to stay away from the areas when planes are spraying, which is done weekly.

Labor on banana farms is either hired or family labor. Most producers in Magdalena hire labor (61.9%) and a large share of them use family labor (44.3%), with 21% of producers using a mix of family and hired labor. About one in six (17.8%) family laborers are women. Producers in Antioquia rely on hired labor. A small share (7.9%) of banana producers works by themselves. This highlights the differences in banana labor in the departments since producers in Antioquia use only hired labor, whereas those in Magdalena also rely on family.

3.1.4 Seeds

Understanding the source of seeds is crucial, especially considering that TR4 can be transmitted through infected soil. In Magdalena, the majority of producers (65%) primarily use corms as their source of banana seeds, followed by seedlings (35%). The majority of them independently source seeds, often taking them from other plots (79%). A smaller percentage opts to purchase certified seeds (20%), and 1% receive seeds as gifts. In contrast, all producers in Antioquia plant seeds from their own plots. Additionally, 6.7% of Antioquia's producers choose seedlings, while 93.3% prefer corms as their primary seed source. The differences in the use of seedlings

and corms between producers in Magdalena and Antioquia are statistically significant at the 5% level (see Table A1). Regarding awareness of ICA-certified establishments for banana seed sale, just over half of producers in both Magdalena and Antioquia were aware of these establishments.

3.1.5 Yields and markets

Banana production in the study area primarily targets export markets, although bananas that do not meet international requirements are also sold domestically. All producers in both departments engage in international market sales. Banana farmers can receive higher prices when certified. Rainforest Alliance, Global G.A.P., and Fairtrade are the most common certifications that banana farmers acquire. All 15 producers surveyed in Antioquia have at least one certification, whereas in Magdalena, 79.89% of producers are certified. The yield data, presented in Table 2, reflects a higher yield in Antioquia (44.1 tons/ha) compared to Magdalena (38.66 tons/ha), which aligns with the expectation that producers in Antioquia have better access to technology.

Table 2. Yield in 2022 (tons per hectare) by department (N = 171)

Department	Mean	Median	Standard deviation	Minimum	Maximum
Magdalena (N = 160)	38.66	40.00	15.29	10.4	80.4
Antioquia (N = 11)	46.24	44.10	13.68	31.92	75.47

Source: Own elaboration based on producer survey.

A large majority of the harvest is destined for export, with producers from the two departments selling on average 95% of the harvest to export markets The remaining 5% is either sold domestically or utilized for household consumption by the producers themselves. Bananas that fail to meet export standards, referred to as discarded bananas, are sold in local markets at reduced prices. Income data from nine producers in Antioquia and 144 in Magdalena indicate

a significant disparity in average gross income from banana sales in 2022. Producers in Antioquia report an average income of around $2 million USD, while those in Magdalena earn an average of $143,000 USD, highlighting distinct socioeconomic conditions between the two regions.

3.1.6 Membership in associations/cooperatives

In Colombia, producer associations and cooperatives play a crucial role in organizing and supporting banana producers in selling their products to international markets. A high share of the sampled producers are members of such associations, accounting for 80% in Antioquia and 85.23% in Magdalena. Membership in cooperatives offers several advantages, including the ability to secure better prices, facilitate transactions with other producers, access technical assistance, purchase inputs at reduced costs, and negotiate improved prices for bananas destined for international markets. The semi-structured interviews also revealed that cooperatives are instrumental in the efforts to prevent TR4 from entering the area. These institutions provide biosecurity kits, including footbaths, back pumps, pressure washers, full-body clothing, and five pairs of boots, incurring an approximate cost of $688 USD. The financial support for these initiatives is derived from a partnership between banana producer associations and the Colombian government.

3.1.7 Access to technology

Access to information and technology exhibits variations in the study area. The majority of producers in Magdalena own a smartphone (80%), but there is a connectivity gap, with 24% lacking internet access. Among those with internet access in Magdalena, 50% use a combination of mobile data and Wi-Fi, 28% use only mobile data, and 22% use Wi-Fi. In Antioquia, all respondents have smartphones and internet access, indicating a higher level of connectivity. Of these, 60% access the internet using both mobile data and Wi-Fi, while the remaining 40% rely solely on Wi-Fi networks. In Magdalena, internet access is primarily

through personal smartphones (99.3%), followed by personal computers (27.8%), public computers (1.5%), and tablets (0.7%). In Antioquia, all respondents access the internet through their smartphones, and 73.3% also use personal computers.

3.2 Monitoring strategies for TR4

Significant differences exist between overall awareness of and access to training on TR4. In Antioquia, all 15 producers are aware of TR4, with 14 having received training on the topic. These producers were trained an average of eight times on TR4 in 2022. Conversely, in Magdalena, 174 out of 176 banana producers are aware of Fusarium TR4, and 161 of them participated in an average of 5.8 training sessions on TR4 in 2022. The primary providers of these crucial trainings were producer associations (59.6%), the Colombian Agricultural Institute (ICA, 52.8%), and marketing companies (20.5%). In Antioquia, extension agents trained a higher proportion of producers compared to Magdalena, where producer associations and cooperatives were the main providers of training.

Differences exist regarding monitoring (or surveillance) strategies. In Antioquia, all producers have implemented monitoring measures, with 93% conducting symptom monitoring either independently, with the assistance of farm workers, or through hiring someone. Additionally, 1.3% use mobile apps during the monitoring process, and 27% rely on visits from extension agents. In Magdalena, 89% of respondents have implemented monitoring strategies, with 58.6% conducting symptom monitoring through direct involvement or with the assistance of farm workers, 39% relying on extension agents, and 5% utilizing mobile applications. One producer in both Antioquia and Magdalena uses drones for symptom monitoring. Cooperatives and marketing companies play a role in surveillance by supporting producers in the supervision

of banana plantations, as these institutions send technicians to monitor the phytosanitary status of farms.

The frequency of monitoring activities varies based on the strategies employed. When symptom monitoring is conducted by producers or farm workers, it occurs daily as part of the farm's routine activities. In contrast, monitoring conducted by extension agents or drones typically occurs monthly. Furthermore, 79% of the cost for monitoring by extension agents is financed by cooperatives, associations, government institutions, or marketing companies. Regarding prevention strategies to safeguard banana farms from TR4, 100% of producers in Antioquia and 98% in Magdalena have implemented such measures. The implemented strategies are described below.

3.2.1 Disinfection stations and foot baths

Disinfection stations play a crucial role in controlling the spread of TR4. Typically located at the farm's entrance, these stations consist of two zones separated by a cement bench. The first zone serves as the “dirty zone” where individuals leave their regular shoes, while the second zone is the “clean zone.” To leave the disinfection station, one must pass through a footbath with a disinfectant solution. The implementation of these stations ensures that visitors and farm workers do not inadvertently carry the fungus onto farms. Remarkably, all respondents in Antioquia have built and implemented disinfection stations, whereas 77% have in Magdalena. The difference is statistically significant at the 1% level (see Table A1) Many disinfection stations of small-scale farmers were paid by cooperatives. In addition, some inputs, such as quaternary ammonium and boots, were supplied by associations and ICA. Foot baths to disinfect shoes before and after entering the farm are another strategy. Foot baths contain a mix of ammonium quaternary and water. They are strategically placed in various zones of the farm, particularly at different entrances. This additional layer of protection helps eliminate any

potential contamination of the farm's soil. In Antioquia, 73% of respondents have foot baths, which is slightly higher than in Magdalena (69%).

3.2.2 Cement paths and fences

Other strategies aim at building physical barriers. One of these is cement paths, which are built between banana plots to help minimize soil-to-plant contact, thereby reducing the risk of disease transmission. Cement paths minimize direct contact between soil and footwear, tools, vehicles, and animals. They prevent workers or visitors from direct soil contact within banana farms, thus avoiding soil movement and preventing the unintentional spread of contaminated soil on footwear or tires. In Magdalena, 5% of respondents have built cement paths while 33% have in Antioquia: This difference is statistically significant at the 1% level (see Table A1) Fences are very helpful for producers since they prevent animals and people from walking through multiple plantations, which could spread TR4.

3.2.3 Use of technology for managing TR4

Mobile phones, especially smartphones, are the primary means of internet access for producers in both departments. Producers rely on mobile phones to access information about banana production. Specifically, producers use the internet to access various types of agricultural management information (66%), input information (53.5%), and to seek guidance on pests and diseases (42.4%). Fusarium is the most searched disease (76.2% of producers searched for it in the past year), followed by Black Sigatoka (59.5%). According to extension agents and service providers involved in the banana value chain, technology is useful for TR4 prevention when it is used to provide “timely and accurate responses” and “accelerate the protocols for early symptoms”. Further, extension agents believe that technology will be useful in the future by providing satellite spectrums with accurate location and timely information and by shortening detection times for precise information about the crop and location of outbreaks or alarms. In terms of TR4 management, extension agents agree that technology is key for obtaining

"precision and follow-up information", "creating new varieties resistant to the fungus", and "improving the quality of the information". They also mentioned that technology will be more useful in the future by "shortening time for early detection" via "better identification of symptoms" with "apps useful to detect, inform, and locate." Other types of actors interviewed had similar opinions. For example, cooperative managers and government officials viewed the importance of technology for the management of TR4 in terms of obtaining and sharing information, while researchers mentioned that the main role of technology both now and in the future has to do with the development of new banana varieties and improving disease detection times.

The use of mobile phone apps for crop management is much lower than the use of chat groups. A relatively small share of the total sample (16%) utilize apps for crop management, with a much larger share utilizing such apps in Antioquia (60%) compared to Magdalena (11.35%). Some commonly used apps in Magdalena include Avanza, Banasoft, Excel, Farmers Edge, Field Climate, Georeferencing and GPS, Planty, Silver Tracker, and WhatsApp. In Antioquia the apps Xioma and Tropimovil are the most common. These apps primarily serve the purpose of obtaining information about agricultural management, market trends for bananas, and inputs. Banana producers in both departments actively engage in chat groups to exchange valuable information about bananas. In Antioquia, 60% of banana producers actively participate in such chat groups, with all of them using WhatsApp and 22% using Facebook groups. Similarly, in Magdalena, 48.3% of respondents participate in similar groups, with all using WhatsApp and just 2.3% using Facebook or 1.2% Telegram. These results show that banana producers connect with one another and seek advice through their mobile phones.

3.3 Cost benefit analysis (CBA) of implementing TR4 biosecurity measures

A CBA was conducted to assess the benefits and costs of implementing biosecurity strategies aimed at preventing the spread of TR4. The CBA evaluates both costs (expenses) and advantages (benefits). As explained in Section 3, the analysis was conducted per hectare, applying a 40-year time horizon, 12% social discount rate, and an annual depreciation rate of 2.22% and 2.5% for disinfection stations and cement paths, respectively. Below, we present a detailed breakdown of the CBA analysis.

3.3.1 Costs estimation of TR4 disinfection stations, footbaths, and cement paths

Data from surveyed producers is used to estimate the construction costs of disinfection stations and cement paths. These costs included initial construction expenses, annual depreciation, regular maintenance, and necessary operational inputs (such as disinfectants, boots), and supervision. The costs of cement paths were calculated based on an average of 1.3 meters of cement paths per hectare. This estimation is derived from the information provided by producers who have implemented this strategy. Table 3 a comprehensive overview of the costs per hectare associated with the establishment of disinfection stations and cement paths in Magdalena and Antioquia. Given that Antioquia boasts large-scale producers and Magdalena predominantly consists of small and medium-sized producers, this disparity in costs can be explained by the fact that the costs per hectare tend to decrease with increases in farm size. For example, maintenance costs are lower per hectare in Antioquia because they can buy inputs in bulk, whereas smaller farmers in Magdalena face higher costs per hectare.

Table 3. Costs of disinfection stations and cement paths (in USD per hectare)

	Magdalena (N = 73)	Antioquia (N = 6)
Disinfection stations		
Construction (setup costs in year 0)	$396	258.0
Depreciation (annual)	$9	$5.7

Maintenance (annual)	$119	$128.6
Supervision (annual)	$1,863	$1,647.1
Water (annual)	$0.03	$2.21
Quaternary ammonium cost (annual)	$59.61	$673
Boots (annual)	$35.6	$39.3
Cement paths		
Construction (setup costs in year 0)	$98	$28.50
Depreciation (annual)	$2	$0.70
Maintenance (annual)	$4	$1.10

Source: Own elaboration based on producer survey.

3.3.2 Benefits as avoided losses: Exclusion benefits

As detailed in the methods section, benefits were computed as the avoided losses—referred to as exclusion benefits—resulting from the implementation of biosecurity strategies, namely disinfection stations and cement paths. Utilizing data obtained from banana producer surveys, the average yield per hectare was calculated for each region (Magdalena and Antioquia) and multiplied by the average selling price of bananas received by each producer in the respective regions. This calculation provided an estimate of the income that would be preserved due to the adoption of these strategies. Table 4 presents information on average banana yields, the value of bananas per ton, and the substantial financial benefits arising from the implementation of biosecurity strategies in Magdalena and Antioquia. In Magdalena, the average yield is 38.66 tons of bananas per hectare, while in Antioquia, the average yield is higher, at 46.24 tons of bananas per hectare. This implies that Antioquia, with its higher yield per hectare, attains greater financial benefits from these strategies compared to Magdalena, even though producers in this zone receive a lower average price for their production than those in Magdalena.

However, it is noteworthy that these differences between the yield and prices of each region are not statistically significant (see Table A1)

Table 4. Agricultural yield and economic benefits by department (N = 183)

Cost per hectare	Magdalena (N = 169)	Antioquia (N = 11)
Yield (tons/ha)	38.66	46.24
Price per ton of bananas	$395.9	$355.2
Total benefits (avoided losses per hectare)	$15,305.49	$16,424.45

Source: Own elaboration from producer survey.

3.3.3 Net present value and benefit-cost ratio (BCR)

To calculate the NPV and BCR, we combined data from several sources. As producers were reluctant to disclose cost information, we relied on data from the Ministry of Agriculture and Rural Development (2017), indicating that the annual expenses for banana production in Colombia amount to $2,184 (USD/ha). We adjusted this figure for inflation, considering that our data was collected in 2022, resulting in a cost of $2,854 (USD/ha) in Magdalena. Notably, large producers in Antioquia exhibit, on average, 13.2% higher production costs than small producers in Magdalena (Martínez & Peña, 2006). Accordingly, we estimate the cost of production per hectare in Antioquia to be $3,230 (USD/ha). This discrepancy in production costs between the two departments persists, as confirmed by an independent expert specializing in banana production in Colombia. Production costs in Antioquia are elevated due to the increased expenses associated with aerial spraying of Black Sigatoka, a banana plant disease more prevalent in Antioquia than in Magdalena, attributable to higher rainfall in the former.

Table 1 summarizes the financial performance metrics of the biosecurity strategies implemented in banana farms in Magdalena and Antioquia. As elaborated in the methods section, NPV was calculated by subtracting present costs from present benefits. In evaluating

the financial implications of implementing biosecurity strategies, namely disinfection stations and cement paths, to counter the spread of TR4 in banana farms, both regions demonstrate positive NPV per hectare, indicating that the implementation of biosecurity strategies on banana farms to prevent the spread of Fusarium TR4 is a very favorable investment. In Magdalena, the NPV per hectare of these investments is $95,389 USD, while Antioquia records a slightly higher NPV of $112,527 USD. These very high NPV values indicate that the benefits derived from implementing these biosecurity strategies outweigh the initial costs, and that the investments are expected to generate favorable returns over time. Notably, Antioquia's marginally higher NPV aligns with the presence of large banana producers who also exhibit higher yields than in Magdalena. This economic perspective underscores the importance of adopting and sustaining these strategies to safeguard the future of banana cultivation in both regions. Comparing the NPV of investments to the cost of these investments shows that the costs of investing in mitigation methods are much lower than the benefits they could provide.

Table 5. Net present value and benefit-cost ratio by department (N = 183)

	Magdalena	Antioquia
Net Present Value (NPV)	$95,389	$112.527
Benefit-cost ratio	3.1	4.2

Source: Own elaboration based on producer survey.

A BCR greater than 1 indicates that the investment will generate more benefits than costs. The BCR in both departments is greater than 1. In the case of Magdalena, the expected return on investment is particularly noteworthy, with a BCR of 3.1. This implies that each unit of investment in both banana production and biosecurity measures to prevent TR4 (disinfection station, disinfection of footwear and cement paths) is expected to generate 3.1 units of benefits, i.e., the benefits are nearly three times greater than the costs. Similarly, in Antioquia, the financial outlook is also very favorable, with an BCR of 3.9. These robust returns underscore

the substantial financial advantages biosecurity strategies offer to banana producers, regardless of farm size.

3.4 Economics losses calculated from different dispersion scenarios

We simulated the annual economic losses due to TR4 across four hypothetical dispersion scenarios. This allows us to have a more precise and quantitative view of the possible economic consequences of this disease on the local banana industry. The data shown in Table 6 provides an overview of the potential annual consequences of the spread of Fusarium in Magdalena and Antioquia, in four different dispersal scenarios. The different scenarios entail differences in the rate of dispersal of TR4 affected areas (25%, 50%, 75%, and 100%), which, in turn, affect agricultural production, sales, and employment. These factors are used to calculate the potential economic losses resulting from banana production that is no longer sold, because the costs presented do not include those of eradicating plants infected with TR4 or other indirect costs as result of the TR4 presence in Colombia. The predicted outcomes are based on the annual yield data in each department and the most common sale price of a box of 20kg of bananas ($7 USD), which is equivalent to $2.86/kg. We assume all production is exported. The results presented are for bananas only and do not include plantains.

Table 6. Potential annual consequences on bananas by different dispersal scenarios

	Affected area (ha)	Potential annual lost production (tons)	Potential employment losses (number of people)	Potential economic losses per year (USD)
<u>*Magdalena*</u>				
Moderate dispersion (25%)	4,512	178,655	3,609	$62,529,306

Accelerated dispersion (50%)	9,024	357,310	7,219	$125,058,612
Accelerated dispersion (75%)	13,535	535,965	10,828	$187,587,918
Total dispersion (100%)	18,047	714,621	14,438	$250,117,224
Antioquia				
Moderate dispersion (25%)	9,874	325,935	7,899	$114,077,334
Accelerated dispersion (50%)	19,748	651,870	15,799	$228,154,668
Accelerated dispersion (75%)	29,622	977,806	23,698	$342,232,002
Total dispersion (100%)	39,496	1,303,741	31,597	$456,309,336

Source: Based on dispersion scenarios and data from the producer survey.

The findings indicate that even under moderate dispersion, significant losses are evident. In the context of Magdalena, the scenario of moderate dispersal (25%) already entails substantial losses, with thousands of hectares eradicated and significant tonnage of produce lost. However, as the pace of dispersal accelerates to 50%, 75%, and ultimately 100%, the consequences become overwhelming. In the full dispersal scenario, over 18,000 hectares of crops would be lost, accompanied by a loss of more than 700,000 tons of annual production and employment opportunities for over 14,000 individuals. Furthermore, annual economic losses could reach US$250 million, constituting 33.7% of the agricultural GDP of Magdalena and 6.2% of the gross regional product of the department (DANE, 2023).

Similarly, in the case of Antioquia, the dispersal of TR4 would generate economic consequences for the region. In the scenario of moderate dispersion (25%), involving over

9,000 hectares of land cleared of TR4, the outcome includes the loss of thousands of tons of produce annually. This alone poses a substantial setback to the local economy, impacting both agricultural income and employment opportunities. As TR4 dispersal intensifies to 50%, 75%, and complete dispersal (100%), the consequences worsen, with nearly 40,000 hectares eradicated and over 1.3 million tons of annual production lost in the worst-case scenario. The potential economic losses could amount to $456 million USD annually in the worst-case scenario, posing a severe threat to local businesses and impeding overall economic growth in the region. Such a loss represents 15.4% of the agricultural GDP of the department of Antioquia (DANE, 2023).

4. Discussion

This study underscores the importance of preventing the spread of TR4 and highlights the substantial benefits derived from investments in TR4 mitigation methods. The findings underscore that interventions supporting prevention strategies can yield significant positive impacts on the banana sector, potentially preventing substantial economic losses. TR4 poses a serious threat to the banana industry worldwide as it affects the Cavendish variety. By understanding the current state of banana farms, monitoring strategies, the financial viability of mitigation methods, and the economic impact if TR4 were to spread, stakeholders can better design strategies to mitigate the effects of TR4. In this section, we discuss the main results based on the four research questions before discussing the study's limitations and providing policy recommendations.

4.1 Vulnerability of banana farming systems and households to TR4

Despite variations in farm size and labor across both departments, banana farms and associated value chain actors in both Antioquia and Magdalena are highly susceptible to the spread of TR4. The rapid and devastating impact of TR4 on infected soils poses a significant threat.

Common agricultural practices involve the use of corms from existing plots for replanting or establishing new plantations. It is crucial to acknowledge that corms can carry pathogens, including TR4. If the corms used as seeds are infected, they have the potential to introduce these pathogens to new areas or facilitate their transmission within an existing plantation. This scenario can lead to the swift dissemination of this destructive disease.

For most producers, farming is their primary occupation, so they would need to find off-farm work to support their families if TR4 were to spread to their farms. The current food insecurity of farmers in Magdalena would likely be worsened if TR4 were to spread on their farms. The economic dependence on bananas and plantains as primary sources of employment and sustenance creates a vulnerable social, health, and economic fabric. The massive loss of jobs due to plantation closures would lead to an unemployment crisis, leaving numerous families without income, thereby hindering access to food. The resulting scarcity of bananas and plantains in the local market would further worsen the situation, making these products less accessible. The economy's heavy reliance on banana exports would take a substantial hit due to reduced exports. Ultimately, these consequences would create economic and social crises in the region, negatively impacting the quality of life for the local population and leading to a scenario of significant financial hardship.

4.2 Current monitoring strategies and their financial viability

Proactive prevention strategies demonstrate the dedication and awareness of banana producers in Antioquia and Magdalena in safeguarding their livelihoods and banana industry against TR4. Proactive measures adopted by farmers include surveillance of banana plants, building disinfection stations at the entrances, disinfecting tools, machinery, and equipment used on farms, and building cement paths in farms. Different actors from the public and private sector are collaborating to support banana producers in their efforts to combat TR4. It is important to

carry out these measures as they are the main means by which the effect of TR4 can be mitigated, given that there are currently no banana varieties that are resistant or tolerant to TR4. There are statistically significant regional differences in the adoption of some measures, such as disinfection stations and cement paths, which is likely due to differences in purchasing power since there is awareness of TR4 and the importance of disinfection in both regions.

4.3 Benefits greatly outweigh the costs for mitigation methods

By evaluating the costs and NPV associated with investing in TR4 biosecurity measures, the CBA results underscored the substantial benefits derived from the implementation of such measures. Comparing the benefits to the costs, the BCR was estimated to be 3.1 and 3.9 in Magdalena and Antioquia, respectively. Our localized CBA results in Magdalena and Antioquia closely align with the findings of previous studies, with a positive NPV and a high BCR, reflecting the outcomes demonstrated by Scheerer et al. (2018a) and Staver et al. (2020). This consistency between our localized results and broader studies reinforces the viability and effectiveness of TR4 mitigation strategies.

Beyond the raw financial numbers, these strategies promise to make substantial contributions to the prevention of TR4 infection, which stands as an imminent threat to the very heart of these banana-producing regions. It is not only banana producers who are dependent on the banana sector: Local businesses, such as restaurants, drugstores, grocery stores, transportation, motorcycle taxis, agricultural input stores and others, have been created around banana production. The importance of implementing mitigation methods goes beyond their immediate economic benefits. By mitigating the risk of TR4, these strategies help ensure the continuity of banana growing practices, preserving not only the livelihoods of producers, but also the economic backbone of these communities. This resilience, in turn, translates into economic stability for the regions, fostering employment opportunities and maintaining the supply of this

essential agricultural commodity. Therefore, the implementation of these strategies represents a multifaceted approach that harmonizes economic and social aspects, reinforcing the long-term sustainability of the banana industry in Magdalena and Antioquia given that implementation reduces the likelihood of finding TR4 in plantations, avoiding negative consequences that last for decades.

4.4 Dispersion scenarios predict large economic losses from TR4

The estimated economic losses from the four dispersion scenarios in Magdalena and Antioquia show that consequences of TR4 could be immense. Depending on the dispersion of TR4 (ranging from 25% to 100%), losses are estimated to be between $63 and $250 million USD annually in Magdalena and between $114 and $456 million USD in Antioquia. These impacts would not only be felt in the banana value chain, but throughout the entire economy. These results are aligned with the opinions of actors in the value chain such as extension agents, cooperative managers, and government agents who are concerned that TR4 might affect their food security and the regional availability of bananas, negatively influence the environment of banana-producing areas, and reduce the income and subsistence of all the people involved in the value chain.

5. Conclusions

Before concluding with recommendations, it is crucial to acknowledge the limitations of this study. Firstly, our focus was specifically on the socio-economic impact of TR4 on banana producers, and as a result, the study did not extend to analyzing plantain production—a crop also affected by TR4 and of significant economic and food security importance in Colombia. Difficulties in gaining the confidence of producers with TR4-affected banana plants contributed to the absence of information on containment practices and associated costs in our sample. Regarding the CBA study, the surveyed banana producers demonstrated limited

knowledge regarding the costs linked to the implementation of biosecurity strategies. Consequently, the CBA could not integrate the costs related to disinfecting tools and machinery. These limitations underscore the necessity for additional research and data collection to improve the precision and comprehensiveness of future assessments in this domain.

Based on the results and discussion, we provide several recommendations to help combat the spread of TR4 in Colombia. These recommendations are also applicable in other countries, such as Venezuela and Peru, where banana production is under threat from TR4.

First, all banana farms should build and utilize cement paths and disinfectant stations. Since TR4 is a soil-borne pathogen, disinfection stations strategically located at the entrance of banana farms are a critical line of defense: They not only prevent the introduction and transmission of the pathogen, but also help contain its spread through the rigorously disinfection of footwear in direct contact with the soil. Disinfectant stations should not only be built and used for footwear, but also for machinery and vehicles moving between farms. Support for building disinfectant stations and cement paths should be provided by policymakers and investors. Given that marketing companies currently support banana producers against other diseases, they may also be interested in also supporting banana producers in combating TR4. Despite some producers wanting to implement biosecurity measures, it is not feasible for them due to income constraints. For this reason, we recommend that the government should incentivize the application of biosecurity measures. In addition, the government, cooperatives, and associations could also provide more support to banana producers in obtaining inputs used for production, giving them some relief in their budget constraint and possibly freeing up funds for TR4 mitigation and monitoring measures.

Second, raise awareness about TR4. Non-banana farmers or laborers are unaware of TR4, so they don’t think twice about trespassing on banana plantations to take bananas without undertaking shoe disinfection. Trespassing could be mitigated if community members were made aware of the dire consequences from TR4. Based on the high level of awareness about TR4 among producers because of sensitization campaigns, we recommend that the government should also raise awareness about the high benefits entailed with investing in TR4 mitigation methods as well as the risks of using corms as seeds. Awareness campaigns about the importance of using certified banana seeds would help guarantee the quality of the plant material and reduce the risk of TR4 infection.

Third, the use of technology, such as digital communication channels, mobile apps, and drones, should be expanded to spread awareness and help detect and mitigation the spread of TR4. Digital communication channels, such as WhatsApp, serve as crucial platforms for knowledge sharing and collaboration. These channels may prove useful to reduce the negative consequences of Fusarium since producers may take advantage of these digital tools to share positive and negative experiences of dealing with TR4. Technology has the potential to improve crop management, disease prevention and management, and information access and sharing. Drone usage for surveillance could be low in Colombia because this technology is expensive and relatively new, so it is not considered as an option by most producers. Cooperatives and associations could pool their resources together to increase the use of drones. The government should also conduct monitoring activities, such as via drones, to see if there are any symptoms of TR4 on plantations. Mobile apps, such as Tumaini, that utilize artificial intelligence also hold great potential for detecting TR4 in banana plants.

Lastly, field trials should be expanded to South America to find other solutions to combat TR4, such as banana varieties resistant or tolerant to TR4 and elicitor applications. Thus far, field trails have been conducted in Asia, Africa, Australia, and Europe (cf. Dita et al., 2018; Mintoff

et al., 2021; García-Bastidas et al., 2022; Zhou et al., 2023), so their applicability to South America is unknown.

Producer associations and cooperatives are key in supporting banana producers as they are the closest institution to farmers and support them in different aspects of banana production, such as obtaining less expensive inputs, providing technical assistance, and – recently with TR4 prevention and surveillance – by facilitating the application of biosecurity measures on banana farms. Producer associations and cooperatives should be involved in each of the above recommendations. A multipronged approach is needed to prevent TR4 from spreading further in Colombia. Increasing awareness, expanding currently used approaches, adopting new technology, and finding banana varieties resistant or tolerant to TR4 is imperative. This study has shown that the benefits of investing in mitigation methods largely outweigh the costs.

References

Aquino A, Bandoles G, Lim VA, Abeleda C, Molina A. Economic impact of Fusarium Wilt disease on Cavendish banana farms in Southern Philippines. In: Proceedings of the Consultation-Workshop on the Socio-economic impacts of Fusarium Wilt Disease of Cavendish Banana in the Asia-Pacific Region.. 2013 Nov 12-14. Davao City, Philippines. Bioversity International. Rome, Italy. 44 – 57.

Asociación de bananeros de Colombia (Augura). (2021). Coyuntura Bananera 2021 [Internet]. 2021 [cited 2022 Dec 13] Available from: https://augura.com.co/wp-content/uploads/2022/04/COYUNTURA-BANANERA-2021.pdf

Banco de la República (Banrep). Tasa Representativa del Mercado (TRM – Peso por dólar) [Internet]. [cited 2023 Sep 27]. Available from: https://www.banrep.gov.co/es/estadisticas/trm

Barahona D. Análisis de riesgo de expansión de Fusarium oxysporum ante el cambio climático en Ecuador [Bachelor's thesis]. Universidad Técnica Estatal de Quevedo, 2021.

Buddenhagen I. Understanding strain diversity in Fusarium oxysporum f. Sp. Cubense and history of introduction of “Tropical Race 4” to better manage banana production. Acta Hortic. 2009;828, 193–204. https://doi.org/10.17660/ActaHortic.2009.828.19

Castro Rodríguez R, Rosales Alvarez R, Rahal A. Metodologías de preparación y evaluación de proyectos de inversión pública: con ayuda de planillas parametrizadas. Universidad de los Andes, 2008. http://hdl.handle.net/1992/26227

Cook DC, Liu S, Edwards J, Villalta ON, Aurambout JP, Kriticos DJ., Drenth A, De Barro PJ. Predicting the benefits of banana bunchy top virus exclusion from commercial plantations in Australia. PLoS One. 2012;7(8). https://doi.10.1371/journal.pone.0042391

Cook DC, Taylor AS, Meldrum RA, Drenth A. Potential Economic Impact of Panama Disease (Tropical Race 4) on the Australian Banana Industry. J. Plant Dis. Prot. 2015;122 (5/6), 229–237

Damodaran T, Rajan S, Gopal R, Yadav A, Yadav K, Shukla PK, et al. Successful community-based management of banana wilt caused by Fusarium oxysporum f.sp. cubense Tropical race-4 through ICAR-FUSICONT. J. Appl. Hortic. 2019;21(1): 37 – 41

Departamento Administrativo Nacional de Estadística (DANE). (2023). Data from: PIB por departamento: Anexos estadísticos PIB departamental 2005-2022 preliminar. Cuentas nacionales departamentales. 2023 [cited 2023 Mar 20] Available from: https://www.dane.gov.co/index.php/estadisticas-por-tema/cuentas-nacionales/cuentas-nacionales-departamentales

Dita M, Barquero M, Heck D, Mizubuti ESG, Staver CP. Fusarium Wilt of Banana: Current Knowledge on Epidemiology and Research Needs Toward Sustainable Disease Management. Front. Plant Sci. 2018. doi: 10.3389/fpls.2018.01468.

Food and Agriculture Organization of the United Nations. (2022). Data from: FAOSTAT statistical database. License: CC BY-NC-SA 3.0 IGO. Crops and livestock products. https://www.fao.org/faostat/es/#data/TCL

Food and Agriculture Organization of the United Nations – FAO, n.d. The Food Insecurity Experience Scale. [cited 2022 Dec 12] https://www.fao.org/in-action/voices-of-the-hungry/fies/en/

García-Bastidas FA, Arango-Isaza R, Rodriguez-Cabal HA, Seidl MF, Cappadona G, Segura R, Salacinas M, Kema GHJ. Induced resistance to Fusarium wilt of banana caused by Tropical Race 4 in Cavendish cv Grand Naine bananas after challenging with avirulent Fusarium spp. PLoS ONE. 2022;17(9). https://doi.org/10.1371/journal.pone.0273335

Hort Innovation. (2020). Final Report: Integrated management of Yellow Sigatoka and other banana diseases in Far North Queensland (BA12007 Impact Assessment). Horticulture Innovation Australia Ltd. https://www.horticulture.com.au/growers/help-your-business-grow/research-reports-publications-fact-sheets-and-more/ba12007/

Iguarán A. Bananeros piden al Gobierno reforzar medidas y recursos contra el Fusarium. El Heraldo; 2022. [cited 2023 Jan 12] https://www.elheraldo.co/magdalena/magdalena-bananeros-piden-al-gobierno-reforzar-medidas-y-recursos-contra-el-fusarium.

Limbaga C, Gervacio T, Notarte A, Juruena M, Dennis P, Lindsay S, Molina A. *Integrated management of* Fusarium *wilt of bananas in the Philippines and Australia.* ACIAR Technical Report No. 105, 2023. Australian Centre for International Agricultural Research, Canberra.

Malik TM, Mohamad Roff MN, Rozetia L, Maimun, T. (2013). Socio-economic impact, research, and development, and policy-making/regulatory of Fusarium Wilt on banana in Malaysia. In: Proceedings of the Consultation-Workshop on the Socio-economic impacts of Fusarium Wilt Disease of Cavendish Banana in the Asia-Pacific Region. Davao City, Philippines. 12-14 November 2013. Bioversity International. Rome, Italy

Martínez H, Peña Y. La cadena del banano en Colombia. Una mirada global de su estructura y dinámica 1991-2005. Ministerio de Agricultura y Desarrollo Rural, Observatorio Agrocadenas; 2006. Colombia, Bogotá

Ministerio de Agricultura y Desarrollo Rural (MADR). Evaluaciones agropecuarias municipales EVA. Base histórica de periodo 2016 – 2020. Producción nacional agrícola, Bogotá; 2022

Mintoff SJL, Nguyen TV, Kelly C, Cullen S, Hearnden M, Williams R, et al. Banana Cultivar Field Screening for Resistance to Fusarium oxysporum f.sp. cubense Tropical Race 4 in the Northern Territory. J Fungi (Basel). 2021;7(8). https://doi.org/10.3390/jof7080627

Molano O, Montoya D. Análisis de producción, rendimiento y exportación de banano en los principales países afectados por el hongo Fusarium oxysporum F. sp. cubense (Foc R4T) y recomendaciones para Colombia [Master's tesis]. Universidad Nacional Abierta y a Distancia – UNAD; 2022

Okungbowa FI, Shittu HO. Fusarium wilts: an overview. Environ. Res. J. 2013;6(2), 83-102

Olivares BO, Rey JC, Lobo D, Navas-Cortés JA, Gómez JA, Landa BB. Fusarium Wilt of Bananas: A Review of Agro-Environmental Factors in the Venezuelan Production System Affecting Its Development. Agronomy. 2021;11, 986. https://doi.org/10.3390/agronomy11050986

Pegg KG, Coates LM, O'Neill WT, Turner DW. The epidemiology of Fusarium Wilt of Banana. Front Plant Sci, 2019;10:1395. doi: 10.3389/fpls.2019.01395

Pemsl DE, Staver C. Strategic assessment of banana research priorities. RTB Working Paper 2014-2. Lima (Peru), CGIAR Research Program on Roots, Tubers and Bananas (RTB). 2014;66 p. https://hdl.handle.net/10568/82674

Piraquive G, Matamoros M, Cespedes E, Chacón J. Departamento Nacional de Planeación (DNP). Actualización de la tasa de rendimiento del capital en Colombia bajo la metodología de Harberger. 2018; [cited 2023 Jan 20] Actualización de la tasa de descuento bajo la metodología de Harberguer (dnp.gov.co)

Scheerer L, Staver C, Dita M, Perez Vicente L, Pemsl D. Strategic assessment of banana fusarium wilt research priorities. Lima (Peru). CGIAR Research Program on Roots, Tubers and Bananas (RTB). RTB Working Paper 2018-2. ISSN 2309-6586. 2018a;40p. https://doi.org/10.4160/23096586RTBWP20182

Scheerer L, Pemsl D, Dita M, Perez-Vicente L, Staver C. A quantified approach to projecting losses caused by Fusarium wilt Tropical race 4. In: Van den Berg, I. et al, editors. Proceedings. X International Symposium on Banana: ISHS - ProMusa Symposium on Agroecological Approaches to Promote Innovative Banana Production Systems. Acta Hortic 2018b;1196, p. 211-218. ISBN: 9789462611924. https://doi.org/10.17660/ActaHortic.2018.1196.26

Staver C, Bustamante O, Siles P, Aguilar C, Quinde K, Castellón J, et al. Intercropping bananas with coffee and trees: prototyping agroecological intensification by farmers and scientists. *Acta Hortic*. 2013;986, 79–85

Staver C, Pemsl DE, Scheerer L, Perez Vicente L, Dita M. Ex ante assessment of returns on research investments to address the impact of Fusarium wilt tropical race 4 on global banana production. *Front. Plant Sci.* 2020;11, 844. https://doi.org/10.3389/fpls.2020.00844

Zhou GD, He P, Tian L, Xu S, Yang B, Liu L, et al. Disentangling the resistant mechanism of Fusarium wilt TR4 interactions with different cultivars and its elicitor application. Front. Plant Sci. 2023;14:1145837. https://doi.org/10.3389/fpls.2023.1145837

Appendix A: Supplemental data

Table A1. Mean t-tests for comparing averages between departments

Variable	Stat	Antioquia	Magdalena	Difference
Farm area (ha)	Mean	134.56	12.19	122.37***
	Std. err	(49.71)	(32.04)	-
	N	14	175	-
Plant seedlings (B)	Mean	0.07	0.36	-0.29**
(Yes = 1, No = 0)	Std. err	0.07	0.04	-
	N	15	176	-
Plant corms (B)	Mean	0.93	0.64	0.29**
(Yes = 1, No = 0)	Std. err	0.07	0.04	-
	N	15	176	-
Yield (tons/ha/year)	Mean	46.24	38.66	7.58
	Std. err	4.12	1.2	
	N	11	160	
Heard about TR4 (B)	Mean	1	0.99	0.01
(Yes = 1, No = 0)	Std. err	(0)	(0.01)	-
	N	15	176	-
Number of TR4 trainings received	Mean	8.07	5.81	2.26
	Std. err	1.22	0.48	-
	N	14	159	-
Implemented a monitoring strategy? (B)	Mean	1	0.9	0.1
(Yes = 1, No = 0)	Std. err	0	0.02	-
	N	15	175	-
	Mean	1	0.78	0.22**

Built disinfection station (B)	Std. err	0	0.03	-
(Yes = 1, No = 0)	N	15	174	-
Producer disinfects shoes (B)	Mean	0.73	0.7	0.03
(Yes = 1, No = 0)	Std. err	0.46	0.5	
	N	15	174	
Producer disinfects tool/machinery (B)	Mean	0.2	0.21	-0.01
(Yes = 1, No = 0)	Std. err	0.11	0.03	-
	N	15	174	-
Built cement paths (B)	Mean	0.33	0.05	0.28***
(Yes = 1, No = 0)	Std. err	0.13	0.02	-
	N	15	174	-

Notes: (B): binary variables tested using the Fisher's exact test. Level of significance is: *p < 0.10, **p < 0.05, and ***p < 0.01.

Figure 1

Click here to access/download;Figure;Figure 1.jpg

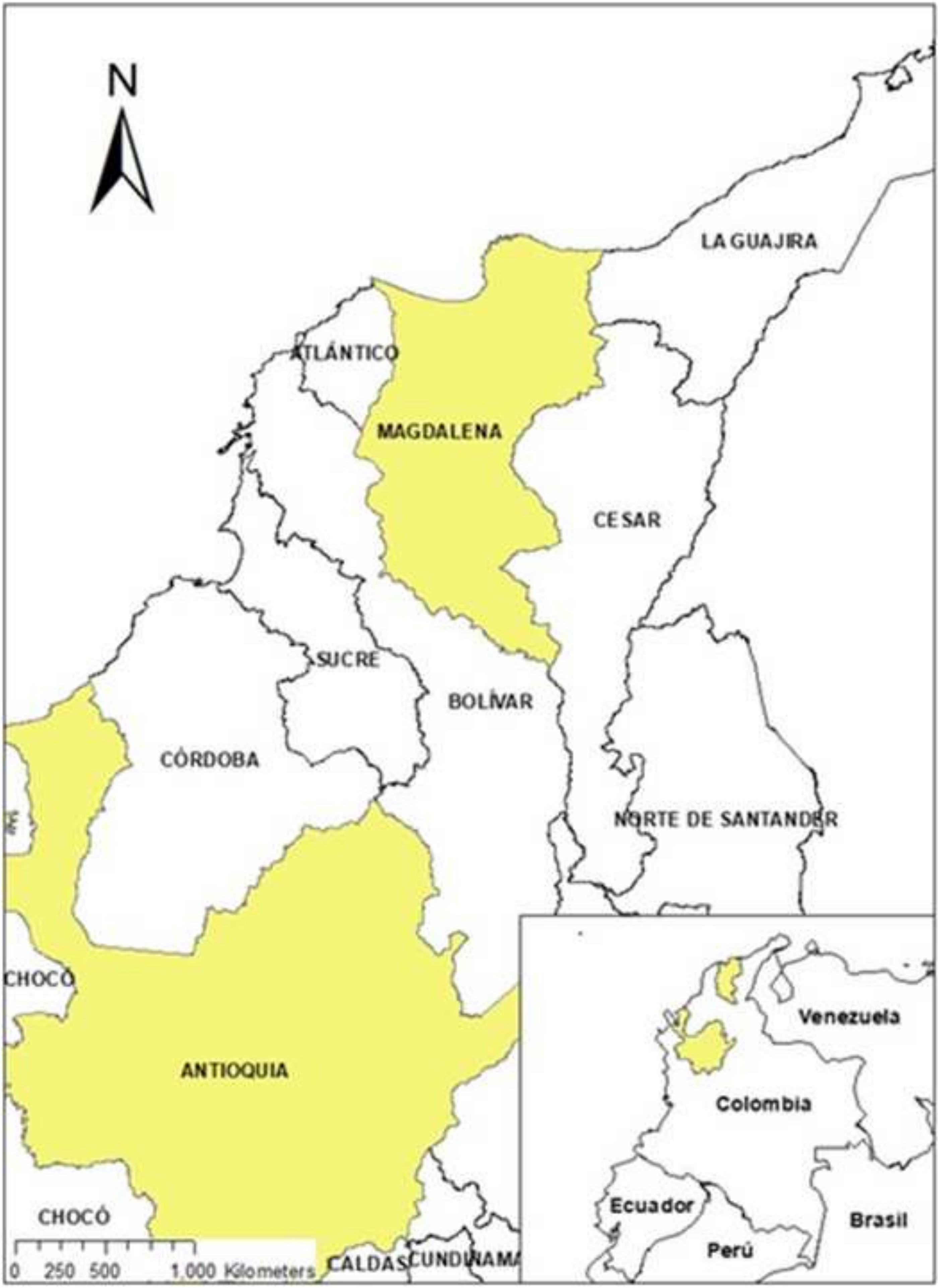

Figure 2

Click here to access/download;Figure;Figure 2.jpg

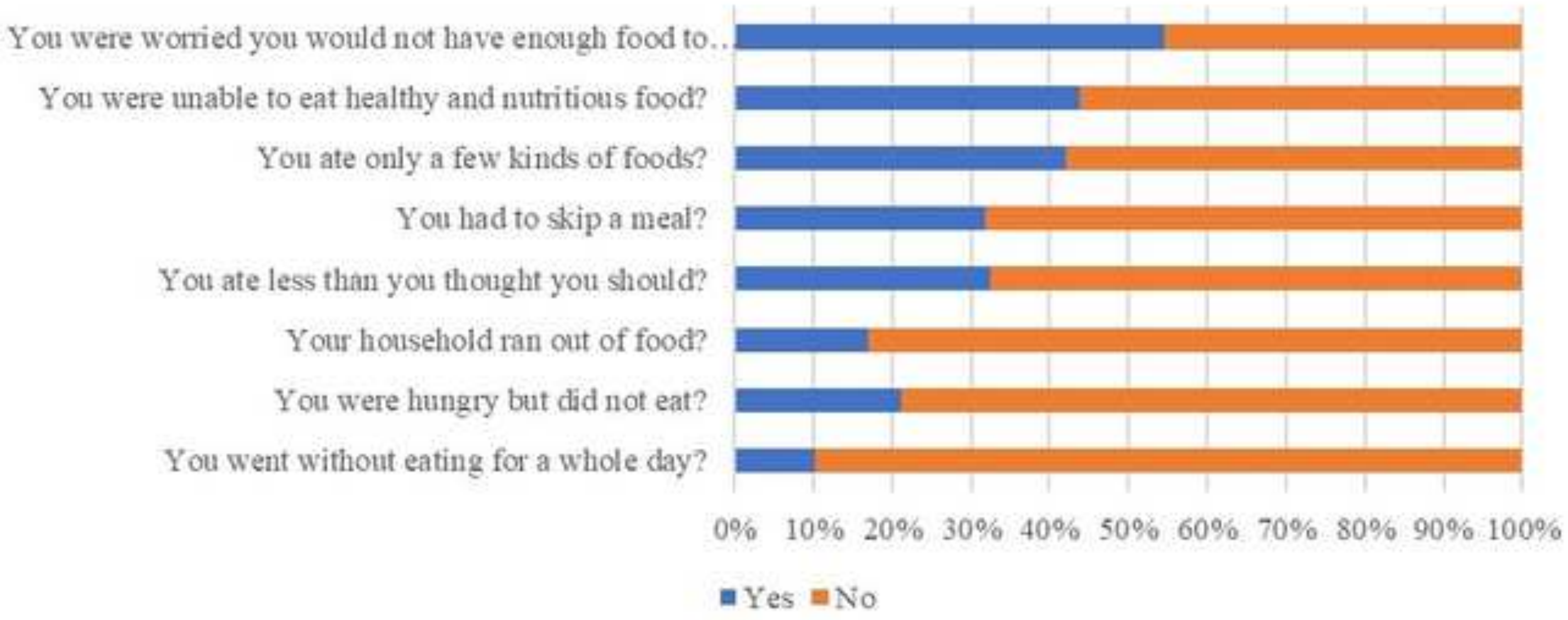

Source: Own elaboration based on the Food Insecurity Experience Score in the producer survey. Antioquia is not shown since all surveyed producers responded "no" to all questions there.
