# Supplementary material for: A socioeconomic and cost benefit analysis of Tropical Race 4 (TR4) prevention methods among banana producers in Colombia
Source: PLoS One. 2024 Oct 30;19(10):e0311243. doi: 10.1371/journal.pone.0311243 (PMC11524470; doi:10.1371/journal.pone.0311243)
Supplement: Attachment — Submitted filename: Reply to reviewers CLEAN.docx [file pone.0311243.s004.docx]

Reply to reviewers

Thank you for the opportunity to revise and resubmit our manuscript. Please find our replies to the editors’ and reviewers’ comments below in blue.

When submitting your revision, we need you to address these additional requirements.

1. Please ensure that your manuscript meets PLOS ONE's style requirements, including those for file naming. The PLOS ONE style templates can be found at

<https://journals.plos.org/plosone/s/file?id=wjVg/PLOSOne_formatting_sample_main_body.pdf> and

<https://journals.plos.org/plosone/s/file?id=ba62/PLOSOne_formatting_sample_title_authors_affiliations.pdf>

Thank you for including these helpful links. We have formatted the manuscript to ensure it meets PLOS ONE’s style requirements.

2. In your Methods section, please provide additional information regarding the permits you obtained for the work. Please ensure you have included the full name of the authority that approved the field site access and, if no permits were required, a brief statement explaining why.

We have added in the Methods section that there were no permits required for field site access (line 195).

3. We note that the grant information you provided in the ‘Funding Information’ and ‘Financial Disclosure’ sections do not match.

When you resubmit, please ensure that you provide the correct grant numbers for the awards you received for your study in the ‘Funding Information’ section.

Thank you. Our grant number is O143. We have added this to the ‘Funding Information’ section.

4. Thank you for stating the following financial disclosure:

"This work was funded by the Deutsche Gesellschaft für Internationale Zusammenarbeit (GIZ) in the project, “Innovations for the prevention and management of the banana fungal disease Foc TR4 (ALER4TA)”, implemented by the Fund for the Promotion in Agriculture (i4Ag) as part of the special initiative Transformation of Agricultural and Food Systems."

Please state what role the funders took in the study. If the funders had no role, please state: ""The funders had no role in study design, data collection and analysis, decision to publish, or preparation of the manuscript.""

If this statement is not correct you must amend it as needed.

Please include this amended Role of Funder statement in your cover letter; we will change the online submission form on your behalf.

Thank you. We have added the sentence suggested above in the cover letter since the funders had no role.

5. Thank you for stating the following in the Acknowledgments Section of your manuscript:

"Special thanks to Jorge Eliecer Vargas who provided valuable guidance and information during the socioeconomic study. We are grateful for the funding for this work from the Deutsche Gesellschaft für Internationale Zusammenarbeit (GIZ). We are thankful for the support in the data collection process to the banana producer associations Augura and Asbama, the producer cooperatives, members from the Latin American and Caribbean Network of Fair-Trade Small Producers and Workers (CLAC, for its acronym in Spanish) and, last but not least, to Leonardo García and his data collection team. The opinions expressed are those of the authors, and do not necessarily reflect those of the funders or institutions of affiliation."

We note that you have provided funding information that is not currently declared in your Funding Statement. However, funding information should not appear in the Acknowledgments section or other areas of your manuscript. We will only publish funding information present in the Funding Statement section of the online submission form.

Please remove any funding-related text from the manuscript and let us know how you would like to update your Funding Statement. Currently, your Funding Statement reads as follows:

"This work was funded by the Deutsche Gesellschaft für Internationale Zusammenarbeit (GIZ) in the project, “Innovations for the prevention and management of the banana fungal disease Foc TR4 (ALER4TA)”, implemented by the Fund for the Promotion in Agriculture (i4Ag) as part of the special initiative Transformation of Agricultural and Food Systems."

Please include your amended statements within your cover letter; we will change the online submission form on your behalf.

We have removed the funding information from the Acknowledgements Section. In addition, we confirm that the funding statement previously submitted is correct.

6. In the online submission form, you indicated that "Not all data can be shared publicly because of concerns of confidentiality. Some data are available from the authors (contact via Jonathan Mockshell at j.mockshell@cgiar.org) for researchers who meet the criteria for access to the data."

All PLOS journals now require all data underlying the findings described in their manuscript to be freely available to other researchers, either 1. In a public repository, 2. Within the manuscript itself, or 3. Uploaded as supplementary information.

This policy applies to all data except where public deposition would breach compliance with the protocol approved by your research ethics board. If your data cannot be made publicly available for ethical or legal reasons (e.g., public availability would compromise patient privacy), please explain your reasons on resubmission and your exemption request will be escalated for approval.

Thank you. We have addressed this in the cover letter.

7. Please amend either the title on the online submission form (via Edit Submission) or the title in the manuscript so that they are identical.

Thank you for pointing out this discrepancy. We have ensured that the titles match.

8. We note you have included a table to which you do not refer in the text of your manuscript. Please ensure that you refer to Table 5 in your text; if accepted, production will need this reference to link the reader to the Table.

Thank you. We now refer to Table 4 (previously Table 5) in the paragraph above it.

9. We notice that your supplementary tables are included in the manuscript file. Please remove them and upload them with the file type 'Supporting Information'. Please ensure that each Supporting Information file has a legend listed in the manuscript after the references list.

Thank you. We have moved the table from the end of the manuscript to a separate “Supporting information” file. We also added a Supporting information legend to the manuscript below the reference list.

**Review Comments to the Author**

**Reviewer #1**:

Scientific Quality:

-The paper presents an important contribution to the literature by investigating the socioeconomic implications of Fusarium oxysporum f. sp. cubense Tropical Race 4 (TR4) on banana producers in Colombia. The study employs a mixed-methods approach, including interviews with producers and key stakeholders, as well as a cost-benefit analysis, to assess the susceptibility of banana farming systems to TR4. The findings shed light on the financial viability of current monitoring strategies and provide valuable insights into the effectiveness of preventive measures implemented by producers.

Thank you for taking the time to read our manuscript and provide valuable feedback and comments. We appreciate your time and dedication to improving our manuscript and thus knowledge base in the scientific community. We have replied to each of your comments below, with line numbers referring to those in the version with tracked changes.

-While the study provides a comprehensive analysis of the socioeconomic aspects of TR4 on banana producers in Colombia, the methodology section lacks detail regarding the sampling strategy for selecting interview participants. Providing information on how participants were chosen would enhance the transparency and reproducibility of the study.

Thank you for bringing this to our attention. We added more detail to the paper in terms of the sampling strategy (lines 214-223). To summarize, all banana producers who visited cooperatives when the enumeration team was present and all banana producers encountered in villages where the cooperative leaders took the enumeration team were asked to participate in the survey. However, about 60% of producers approached in cooperatives and 50% approached in the field declined to participate in our study. Despite explaining the confidentiality of participation, producers still feared that their economic standing and ability to take out loans would be affected through their participation in our study.

- The paper would benefit from a more thorough discussion of the limitations inherent in the cost-benefit analysis. For instance, the analysis may overlook certain intangible costs and **benefits** associated with TR4 mitigation measures, such as the psychological impact on farmers or the long-term environmental consequences. For example:

Line 652: The study of Martínez et al. (2023) and Olivares (2023) underscores the critical threat posed by TR4 to the global banana industry, particularly in Latin America and the Caribbean (LAC), where its presence has been increasingly reported. Indeed, as highlighted by Rodríguez et al. (2023a; 2023b), Campos (2023) TR4 represents a significant risk to banana production in LAC countries, including Colombia, Peru, and Venezuela. The recent emergence of TR4 in these regions underscores the urgency for effective containment and control measures to safeguard the banana industry and mitigate potential economic losses.

Moreover, the socioeconomic characterization of banana-producing regions, such as the Changuinola district in Panama (Rodríguez et al., 2021; Gracia et al. 2020; Montenegro et al. 2021), serves as a valuable reference for understanding the socioeconomic dynamics and vulnerability factors that may exacerbate the impact of TR4 on local communities. Identifying key socio-economic indicators, such as education, unemployment, and food security, enables stakeholders to formulate tailored strategies to enhance resilience and support sustainable land management practices (Olivares et al. 2020; Olivares and Hernández, 2020).

Furthermore, Vega et al. (2022); Olivares et al. (2022); and Campos (2023), emphasize the need for proactive measures, including prevention, rapid containment, and quarantine protocols, to mitigate the spread of TR4 and safeguard the livelihoods of small and medium-sized banana farmers in affected regions. By integrating risk analysis and climatic suitability mapping, stakeholders can identify high-risk areas and implement targeted interventions to prevent further dissemination of the pathogen. The findings presented in the paper underscore the urgent need for coordinated efforts among stakeholders, including government agencies, researchers, and local communities, to combat the spread of TR4 and safeguard the long-term sustainability of the banana industry in Colombia and beyond.

Thank you very much for this very detailed recommendation. Below, we address the points made above.

We have incorporated the aspect of intangible costs and benefits throughout the manuscript, beginning in the methods section where we explain that while CBA can help stakeholders make rational decisions in complex situations, quantifying intangible benefits, such as through an index or monetizing intangible benefits, is difficult and subjective (Kumar et al., 2021; Wun et al., 2022) (lines 230-236). In an attempt to capture these intangible aspects that the cost-benefit analysis is unable to (Hamundu et al., 2012), producers were asked about the impact of TR4 on different aspects of their lives and community, namely on the economy, jobs, food security, and the environment. Words which respondents used highlight some of the intangible impacts of TR4. We did not include this in the previous version of the manuscript, but have now added this qualitative information to the revised version. These qualitative results supplement the quantitative CBA (lines 280-287) and the results are now presented in a new subsection “Intangible aspects of TR4” (lines 766-778), which is also now discussed in the Discussion section (lines 944-954).

Regarding the cost-benefit analysis, we admit that our analysis is based on partial budgeting which does not fully capture all the costs including the intangible costs and benefits associated with TR4 prevention measures and thus may underestimate the full burden of TR4 (lines 927-928). For example, there are intangible costs of TR4 such as fear, agricultural equity, social inclusion, loss of markets, insecurity, and isolation (Aquino et al., 2012; Pattison et al., 2013) (lines 928-932). In addition, there are intangible benefits of TR4 mitigation, such as increased certainty about banana farming, biodiversity, mental and physical health, reduced public costs for control of future outbreaks, and the cultural and traditional importance for families and communities to continue to banana farm (Wun et al., 2022) (lines 932-937). We now discuss these at the end of the subsection “Limitations of the study” towards the end of the Discussion section.

Despite these limitations, we used different methods in the estimation of the cost-benefit analysis which shows consistent results. We also acknowledge that valuing intangible costs and benefits can be extremely difficult. It is recommended that future studies include methodologies that allow these intangible aspects to be analyzed, identified and included. This suggestion was added in “Future research opportunities” section of the manuscript (see lines 962-967).

We have now included the studies you mentioned that highlight the risk of TR4 to LAC countries in the second paragraph of the manuscript (namely, Campos, 2023; Martínez et al., 2023; Rodríguez et al., 2023a; 2023b, Olivares Campos, 2023) (lines 95-96 and 101-104).

Your example and the literature you discussed on the risk of TR4 in Latin America was added to the discussion in the subsection “Vulnerability of banana farming systems and households to TR4” (lines 773-812).

We added a discussion of other strategies identified and recommended in the literature for the management of TR4 in the subsection “Current monitoring strategies and their financial viability” (lines 813-864).

We incorporated the importance of understanding key socioeconomic dynamics and vulnerability factors (Montenegro et al. 2021; Olivares et al., 2020; Rodríguez et al., 2021) (lines 933-948) and the urgency for effective containment measures in the Conclusion section (lines 973-977).

We liked the second to last and last sentence of your above comment so much, that we rephrased them and included them as the first two sentences of our Conclusion.

Thank you again for your very valuable comments and advice!

-The abstract provides a concise overview of the study; however, it could be improved by including specific quantitative results from the cost-benefit analysis to give readers a better understanding of the magnitude of potential losses and benefits associated with TR4 mitigation strategies.

Thank you. We added specific quantitative results to the Abstract, namely the net present value and benefit-cost ratio from the cost-benefit analysis.

Recommendations:

-Consider providing more detailed information on the sampling methodology employed for selecting interview participants, including criteria for inclusion and exclusion, to enhance the rigor and reproducibility of the study.

Thank you for this suggestion. In the initial phase, we met with government officials and experts in Colombia’s banana sector to determine which actors to interview (lines 208-210). To obtain a wide range of perspectives, we decided to interview a variety of stakeholders. Our proposed sample included actors both with and without TR4 on their banana farms (lines 230-231). However, despite several months of trying to interview producers with TR4, we were unable to interview producers (or managers or banana workers) of farms with TR4 due to their unwillingness to participate in the survey (this was elaborated on in the previous version of the manuscript, starting on line 231 in the revised version). Given our response and changes based on this comment and your second comment on the sampling strategy (which we addressed on lines 214-223), we believe that we have provided enough detail now on the sampling strategy.

-Expand the discussion section to include a more thorough analysis of the limitations of the cost-benefit analysis, including potential biases and uncertainties, to provide a balanced interpretation of the findings.

Thank you for this suggestion. In addition to the above-mentioned inclusion of the limitation of the CBA of not including intangible benefits and costs (lines 932-937), we also now include a discussion of other limitations of the CBA in the newly added subsection “Limitations of the study” (starting on line 904). For example, we elaborate that our results should be interpreted with caution given potential biases and uncertainties. In addition, we specify that the full economic impact of TR4 – especially post-infection – was not taken into account. We also acknowledge in the manuscript that future studies should include data from affected farms and would also benefit from longitudinal data. Despite trying for over four months to gather data from producers with TR4 present on their banana farms, we were unable to interview them because they were not willing to share information with us. While we recommend that future studies gather data from these producers, we don’t have any clear advice on how researchers can practically do this given the extreme difficulties we faced interviewing this subset of producers.

Improvement Advice:

-To strengthen the scientific rigor of the study, consider incorporating quantitative measures of the socioeconomic impact of TR4 on banana producers, such as estimating the potential reduction in yields or income losses due to TR4 infection.

Thank you for this very valuable suggestion. We added a table (Table 5, line 735) that includes estimates of the annual impact of TR4 at the department-level. We show impacts on the number of hectares, banana production, direct and indirect employment, export losses, and the loss of land value. In addition, we show impacts at the producer-level in Table 6 (line 745) on affected areas, banana production, export income, domestic sales income, and land value for the average producer in each department.

-To enhance the generalizability of the findings, consider expanding the study to include a broader sample of banana-producing regions in Colombia and possibly other countries affected by TR4, to capture variations in socioeconomic conditions and farming practices.

Thank you for this comment. The two study regions were chosen for very specific reasons: they are the top two banana producing departments in Colombia; they have distinct farming systems so we could compare farming systems; they have different banana markets (export vs. domestic bananas); they mostly plant with Cavendish cultivators to produce export bananas; and one department (Magdalena) had three confirmed cases of TR4 when the study took place (lines 166-179). At the time of data collection, we were not able to conduct the study in other regions of Colombia or in other countries because of funding constraints. We had initially set out to conduct interviews in another department, La Guajira, which had confirmed cases of TR4 on large banana plantations; however, managers of these large plantations were unwilling to speak with enumerators, the authors, and hired consultants, let alone be interviewed, so we had to withdraw La Guajira from our study area. In addition to the already related and cited studies that took place in other countries, we have added the references you cited below to provide more generalizability of our findings. For example, in the Conclusion (lines 979-982) we state that the recommendations could be evaluated and adapted to local contexts in other countries, such as neighboring Venezuela and Peru, where banana production is threatened by TR4 (Campos, 2023; Rodríguez et al., 2023a, 2023b).

References:

-Incorporating the suggested and relevant recent literature is crucial for enriching the scholarly discourse surrounding the socioeconomic impact of Fusarium wilt (TR4) on banana production in Colombia and neighboring regions. By drawing upon the latest research findings and insights from impact journals, such as those focusing on banana pathology, agricultural economics, and regional socioeconomic dynamics, the manuscript can offer a comprehensive and up-to-date analysis of the challenges and opportunities faced by banana producers. Furthermore, integrating recent literature enhances the paper's credibility, ensuring that it aligns with the latest advancements and understanding in the field. This comprehensive approach not only strengthens the scientific foundation of the study but also provides valuable context and perspective for policymakers, researchers, and stakeholders seeking effective strategies to mitigate the impact of TR4 on banana production and safeguard the livelihoods of farming communities.

Campos, B. O. O. (2023). Banana production in Venezuela: Novel solutions to productivity and plant health. Springer Nature. <https://doi.org/10.1007/978-3-031-34475-6>

Gracia, E. J. M., Rodríguez, J. E. P., & Campos, B. O. O. (2020). Adaptation to climate change in indigenous food systems of the Teribe in Panama: a training based on Cristal 2.0. Revista Luna Azul, (51), 182-197. : <https://doi.org/10.17151/luaz.2020.51.10>

Martínez, G.; Olivares, B.O.; Rey, J.C.; Rojas, J.; Cardenas, J.; Muentes, C.; Dawson, C. 2023. The Advance of Fusarium Wilt Tropical Race 4 in Musaceae of Latin America and the Caribbean: Current Situation. Pathogens, 12, 277. https://doi.org/10.3390/pathogens12020277

Montenegro-Gracia, E. J., Pitti-Rodríguez, J. E., & Olivares-Campos, B. O. (2021). Identification of the main subsistence crops of Teribe: a case study based on multivariate techniques. Idesia (Arica), 39(3), 83-94. <http://dx.doi.org/10.4067/S0718-34292021000300083>

Olivares B, Vega A, Calderón MAR, Rey JC, Lobo D, Gómez JA, Landa BB. 2022. Identification of Soil Properties Associated with the Incidence of Banana Wilt Using Supervised Methods. Plants, 11(15):2070. <https://doi.org/10.3390/plants11152070>

Olivares, B., Hernández, R. 2020. Application of multivariate techniques in the agricultural land’s aptitude in Carabobo, Venezuela. Tropical and Subtropical Agroecosystems, 23(2):1-12. <http://dx.doi.org/10.56369/tsaes.3233>

Olivares, B., Pitti, J., Montenegro, E. 2020. Socioeconomic characterization of Bocas del Toro in Panama: an application of multivariate techniques. Revista Brasileira de Gestao e Desenvolvimento Regional, 16(3):59-71. <https://doi.org/10.54399/rbgdr.v16i3.5871>

Olivares, B.O. (2023). Fusarium Wilt of Bananas: A Threat to the Banana Production Systems in Venezuela. In: Banana Production in Venezuela. The Latin American Studies Book Series. Springer, Cham. <https://doi.org/10.1007/978-3-031-34475-6_3>

Rodríguez, J. E. P., Olivares, B. O., Montenegro, E. J., Miller, L., & Ñango, Y. (2021). The role of agriculture in the Changuinola District: A case of applied economics in Panama. Tropical and Subtropical Agroecosystems, 25(1). <http://dx.doi.org/10.56369/tsaes.3815>

Rodríguez-Yzquierdo, G.; Olivares, B.O.; González-Ulloa, A.; León-Pacheco, R.; Gómez-Correa, J.C.; Yacomelo-Hernández, M.; Carrascal-Pérez, F.; Florez-Cordero, E.; Soto-Suárez, M.; Dita, M.; et al. Soil Predisposing Factors to Fusarium oxysporum f.sp Cubense Tropical Race 4 on Banana Crops of La Guajira, Colombia. Agronomy 2023, 13, 2588. https://doi.org/10.3390/agronomy13102588

Rodríguez-Yzquierdo, G.; Olivares, B.O.; Silva-Escobar, O.; González-Ulloa, A.; Soto-Suarez, M.; Betancourt-Vásquez, M. Mapping of the Susceptibility of Colombian Musaceae Lands to a Deadly Disease: Fusarium oxysporum f. sp. cubense Tropical Race 4. Horticulturae 2023, 9, 757. https://doi.org/10.3390/horticulturae9070757

Vega, A.; Olivares, B.O.; Rueda Calderón, M.A.; Montenegro-Gracia, E.; Araya-Almán, M.; Marys, E. 2022. Prediction of Banana Production Using Epidemiological Parameters of Black Sigatoka: An Application with Random Forest. Sustainability 14, 14123. <https://doi.org/10.3390/su142114123>

Thank you for this detailed comment and for providing us with additional references to read and cite in our manuscript. We agree with you that it is important to integrate more current literature on TR4 that has been conducted in a variety of disciplines. In addition to citing all the studies you mentioned below, we dug deeper into the literature to provide more context and generalizability of our findings. For example, related to the disease’s pathology, we added that Foc exhibits diverse and shifting populations of pathogens as a result of changes in environmental conditions (Valverde-Bogantes et al., 2020) and that subtropical race 4 (STR4) can also affect Cavendish under certain abiotic constraints like water logging in sub-tropical regions (García-Bastidas et al., 2022; Roberts et al., 2024) (lines 83-94). We also added how TR4 affects Cavendish and a wide range of local cultivators that are unaffected by R1 and R2 (Ploetz, 2015) (line 89-90). We also added in the discussion that there are trials of tolerant cultivators, including FHIA hybrids and Cavendish clones are being carried out (Blomme et al., 2024 ; Mintoff et al., 2021; Rocha et al., 2021) (lines 830-836). However, there are several challenges to identify market worthy TR4 resistant cultivators, such as bred hybrids like the FHIA lines exhibiting non-ideal fruit characteristics and lower yields (Blomme et al., 2024), as well as Cavendish somaclones which may not meet international markets’ quality standards (Blomme et al., 2024) (lines 834-836). We also added that the literature emphasizes other ways of managing TR4, such as a multi-tiered approach involving the application of integrated pest management and resistant clones (Martínez et al., 2020), phytosanitary geospatial analysis (Pérez-Matos et al., 2023), and susceptibility mapping (Rodríguez et al., 2023) (lines 852-864). Thank you for providing us with the studies cited below, which we used to explore further literature and overall strengthen our manuscript.

**References** (excluding those in your last recommendation above):

Aquino A, Bandoles G, Lim VA, Abeleda C, Molina A. Economic impact of Fusarium Wilt disease on Cavendish banana farms in Southern Philippines. In: Proceedings of the Consultation-Workshop on the Socio-economic impacts of Fusarium Wilt Disease of Cavendish Banana in the Asia-Pacific Region. 2013 Nov. (pp. 44-57).

Blomme G, Kearsley E, Ocimati, W, Dita, M. Fusarium Wilt of Banana: Training manual. Version 1. Bioversity International, Rome, Italy, 2024.

García-Bastidas FA, Arango-Isaza R, Rodriguez-Cabal HA, Seidl MF, Cappadona G, Segura R, et al. Induced resistance to Fusarium wilt of banana caused by Tropical Race 4 in Cavendish cv Grand Naine bananas after challenging with avirulent Fusarium spp. PLoS ONE. 2022;17(9). doi: 10.1371/journal.pone.0273335.

Hamundu FM, Wibowo S, Budiarto R. A hybrid fuzzy-Monte Carlo simulation approach for economical assessment of the impact of ERP technology. Journal of Information and Communication Technology (JICT). 2012; (11):93-111. doi: 10.32890/jict.11.2012.6614

Kumar P, Debele SE, Sahani J, Rawat N, Marti-Cardona B, Alfieri SM, Basu B, Basu AS, Bowyer P, Charizopoulos N, Gallotti G. Nature-based solutions efficiency evaluation against natural hazards: Modelling methods, advantages and limitations. Science of the Total Environment. 2021 Aug 25;784:147058. doi: 10.1016/j.scitotenv.2021.147058

Martínez-Solórzano GE, Rey-Brina JC, Pargas-Pichardo RE, Domínguez M. Actualización sobre el manejo de la marchitez por Fusarium Raza 4 Tropical en musáceas. Agronomía Tropical. 2020; 70: 1-18. doi: 10.5281/zenodo.4291108.

Mintoff SJL, Nguyen TV, Kelly C, Cullen S, Hearnden M, Williams R, et al. Banana Cultivar Field Screening for Resistance to Fusarium oxysporum f.sp. cubense Tropical Race 4 in the Northern Territory. J Fungi (Basel). 2021;7(8). doi: 10.3390/jof7080627.

Pattison A, Lindsay S, O’Neill W, Pearce C, Kukulies T, Wright C. Regulations, Research and Development and Socio-Economic Impact of Fusarium wilt on Banana in Australia. Proceedings of the consultation-workshop on the socio-economic impacts of Fusarium Wilt disease of cavendish banana in the Asia-Pacific region. Davao city, Philippines. 2013; 6-23.

Pérez-Matos AE, González XC, -Villarini RIB, Resto TO, - González GC, González* NA. Fusarium oxysporum f.sp. cubense Tropical Race 4 (Foc TR4): Latent Risk for Banana and Plantains Crops in Puerto Rico. Current Agriculture Research Journal. 2023 Jan 5;11(3):761–80. doi:10.12944/CARJ.11.3.07

Ploetz RC. Fusarium wilt of banana. Phytopathology. 2015;105(12): 1512-1521. doi: 10.1094/PHYTO-04-15-0101-RVW.

Roberts JM, Carvalhais LC, O'Dwyer C, Rincón‐Flórez VA, Drenth A. Diagnostics of Fusarium wilt in banana: Current status and challenges. Plant Pathol. 2024 Jan 22;73(4):760–76 doi: 10.1111/ppa.13863.

Rocha A, Soares J, Nascimento F, Santos A, Amorim, V, Ferreira C, et al. Improvements in the Resistance of the Banana Species to Fusarium Wilt: A Systematic Review of Methods and Perspectives. J. Fungi. 2021; 7:249. doi: 10.3390/jof7040249.

Valverde-Bogantes E, Bianchini A, Herr JR, Rose DJ, Wegulo SN, Hallen-Adams HE. Recent population changes of Fusarium head blight pathogens: drivers and implications. Canadian Journal of Plant Pathology. 2020 Jul 2;42(3):315–29. doi: 10.1080/07060661.2019.1680442.

Wun J, Kemp C, Puett C, Bushnell D, Crocker J, Levin C. Measurement of benefits in economic evaluations of nutrition interventions in low‐and middle‐income countries: A systematic review. Maternal & Child Nutrition. 2022 Apr;18(2):e13323. doi: 10.1111/mcn.13323

**Reviewer #2**:

The article is not solid, the data obtained is hypothetical because the producers interviewed do not have the disease and therefore, there is no direct relationship between the data and the conclusions. For the cost benefit analysis, they used very general cost data that does not come from a reliable source.

Thank you for your feedback on our manuscript. We have replied to each of your comments, with line numbers referring to those in the version with tracked changes.

Your concern about the validity of data from farms without TR4 is understandable. While it is true that we did not include farms already affected by TR4 because they declined to participate in the study (despite many attempts and strategies to try to interview them over a period of four months), the producers included in our study are actively implementing biosecurity measures specifically to prevent the entry of TR4 into their farms. Our cost elements are also based on expert opinions who have several years of experience regarding TR4. This means that the costs and benefits we analyzed are based on actual, ongoing practices, not hypothetical scenarios. We believe that our data comes from a reliable source, namely the banana producers themselves as well as the input from subject matter experts from Colombia. To provide clarity to the reader, a more thorough explanation of the producers interviewed and their experiences with TR4 prevention has been added (lines 214-223 and 230-243). We also added that we confirmed data with subject matter experts (lines 273-276). Lastly, we removed some of the conclusions that were not directly drawn from the data, such as by removing the paragraph on lines 875-889, which was before the subsection “Dispersion scenarios predict large economic losses from TR4”.

The sample size is not adequate, it is unbalanced in the study areas. The data obtained does not allow us to have an analysis of costs, much less the benefits of the implementation of biosecurity vs. the losses that can be caused by the disease that is the focus of the study, TR4. If anything, they can serve to characterize the producers of Magalenda, because they do not even have a sample population that allows them to characterize the producers of Antioquia.

We agree that the sample is not balanced in the study areas; however, this was intentional due to the makeup of producers in the two distinct study areas. The reason for the unbalanced sample is that there are mainly smallholder producers in Magdalena, whereas there are large holders and managers of large farms in Antioquia. Because of this, we were unable to increase the sample size in Antioquia. The two study regions were chosen for very specific reasons: they are the top two banana-producing departments in Colombia; they have distinct farming systems so we could compare farming systems; they have different banana markets (export vs. domestic bananas); they mostly plant with Cavendish cultivators to produce export bananas; and one department (Magdalena) had three confirmed cases of TR4 when the study took place (lines 177-179). At the time of data collection, we were not able to conduct the study in other regions of Colombia or in other countries because of funding constraints. We had initially set out to conduct interviews in another department, La Guajira, which had confirmed cases of TR4 on large banana plantations; however, managers of these large plantations were unwilling to speak with enumerators, the authors, and hired consultants, let alone be interviewed, so we had to withdraw La Guajira from our study area.

Despite the unbalanced data, we are confident that our data allows us to analyze the costs and benefits (as avoided losses, i.e. “exclusion benefits”) of implementing TR4 prevention measures. In addition, we took care to analyze the results by department, so that we did not mix producers from the two distinct study areas. The results and the table in the supplementary material show that there are statistically significant differences between the two study areas. In the revised version of the manuscript, we better recognize and point out the limitations of the study (in a new subsection called “Limitations of the study”, lines 904-939), which include the fact that we were unable to include some key costs and benefits due to data limitations.

The title of the article has nothing to do with the results and furthermore the conclusions reached have nothing to do with the information obtained in the materials and methods, reflecting a very large decontextualization of the TR4 problem in Colombia.

Thank you for this comment. We are unsure if the title you were referring to was one that was incorrectly included in the manuscript file (Fusarium Tropical Race 4 (TR) in Colombia: A dire situation for banana producers and recommendations for combating the disease”) or the title that was in the meta data information (“A socioeconomic and cost benefit analysis of Tropical Race 4 (TR4) among banana producers in Colombia”). Nevertheless, we have revised our title to “A socioeconomic and cost benefit analysis of Tropical Race 4 (TR4) prevention methods among banana producers in Colombia,” which reflects the study and results since the main methods employed are a socioeconomic and cost benefit analysis of TR4 prevention methods among banana producers – not among producers with TR4. While we could not interview producers of bananas with confirmed cases of TR4 despite exhaustively trying for four months to do so, we interviewed banana producers who are aware of TR4 and, for the most part, adopting and implementing monitoring and prevention strategies. The analysis and results were presented and then discussed within the context of Colombia to subject matter experts and other stakeholders. In addition, two of the authors of the paper are Colombian, have studied and worked in the field of agricultural economics and have lived in Colombia their entire lives. They also traveled to the field, visited farms, participated in workshops with banana value chain actors, and engaged with stakeholders and subject matter experts both before and after data collection (to validate our results) at the national and regional levels. They are therefore quite cognizant of the context and importance of TR4 in their home country. Given the above, we do not agree that the paper represents “a very large decontextualization of the TR problem in Colombia.”

Although the authors explain in the text why they do not have information on the farms affected by TR4, the basic information exercise was also very poor, for example, the production costs of bananas for export, if they could be obtained with good work in field and then from this information it would have been possible to estimate the cost of biosecurity implementation.

The study identified the cost of biosecurity measures implemented by producers through a household survey. The most frequently mentioned practices were the installation of disinfection stations for footwear and cement paths. However, for production costs, information from the Ministry of Agriculture was used. This is due to the complexity and competitiveness of the banana sector in Colombia. Initially, the household survey included questions on production costs, but to conduct the survey, we needed approval from the producer associations. Unfortunately, the managers of these associations insisted on the elimination of questions pertaining to production costs, which necessitated our reliance on external data sources for production costs.

The cost of the disease has many dimensions, for example in: loss of jobs, or loss of land value, that were not explored by the authors. On the other hand, it is strange in the document that it was not analyzed what it has cost the country and its institutions to prevent and carry out surveillance on the disease, these data could also be estimated and no effort was made in this regard.

Thank you for this comment. As a result of this comment, we have added Table 5, which estimates losses for each department as a whole and Table 6, which estimates losses by producer in each department. In both tables, we estimate the loss of land value, calculated by using the average cost of renting a hectare of land based on data collected in the producer survey (see Table 5, line 735). The methodology for these tables is described on lines 261-279. We also included the potential loss of employment (both direct and indirect employment) in terms of the number of people who would lose their jobs if TR4 were to spread at different rates and the value of these job losses in Table 5.

In addition to the quantitative data, we also collected qualitative data, which we have added to the revised version of the manuscript. This qualitative data provides more detail on the effects of TR4 on producers (a newly added subsection at the end of the results called “Intangible aspects of TR4”, lines 749-761). In this section, we mention that producers expressed concern about their land and the environmental effects of TR4.

Regarding the cost to Colombia, in the Conclusion section, we added that public and private entities (ICA, Agrosavia, the Ministry of Agriculture, ASBAMA, and AUGRA) in Colombia have agreed to invest over 7.2 million USD in TR4 mitigation, surveillance, and awareness between 2019 and 2023 (lines 994-998). There is no information on how much will be invested in the future.

It is not an article that contributes to knowledge and is not interesting. It does not have rigor in the materials and methods, nor in the recommendations and conclusions, which are not derived from the results obtained, rather they are hypotheses, which also demonstrate a low contextualization and knowledge of the topic and the reality of the disease in Colombia and other South American countries.

Thank you for your comments. We appreciate the opportunity to respond to your concerns and clarify the contributions of our study. Our article provides valuable information in terms of the socioeconomic effects of TR4 and the financial viability of the implementation of interventions used for disease management to prevent the entry of TR4 on banana farms. This is the first study of its kind to do so. As a result of your comments and those from the other reviewers, we have added more detail regarding the data collection process (lines 214-227) and more on the rationale for our methodological choices (such as on lines 208-210). We used a survey design to collect accurate and relevant data, which was based on meeting with stakeholders in a workshop conducted in Magdalena before data collection began, as well as discussions with researchers.

While we did not include producers affected by TR4 because they declined to participate in the study, the producers surveyed actively apply measures designed to prevent the entry of TR4 on their farms. Once data collection was completed, data cleaning was performed, and cost-benefit analysis (CBA) was rigorously conducted using household survey data from producers who actively apply these measures. In addition, the analysis was validated with experts in the field and the banana sector.  This approach ensures that our conclusions are based on real and ongoing practices, rather than hypothetical scenarios. We believe that our findings contribute valuable knowledge to the ongoing efforts to combat TR4 and protect the banana industry in Colombia and beyond.

The recommendations for the prevention of the pathogen indicate a great lack of knowledge of phytopathology in general and of the understanding of the sociocultural patterns of Colombia and Latin America and the Caribbean.

Thank you for sharing your opinion with us. The analysis and results were presented and then discussed within the context of Colombia to subject matter experts and other stakeholders. In addition, two of the authors of the paper are Colombian, have studied and worked in the field of agricultural economics, and have lived in Colombia their entire lives. They also traveled to the field, visited farms, participated in workshops with banana value chain actors, and engaged with stakeholders and subject matter experts both before and after data collection (to validate our results) at the national and regional level. They are therefore very attuned to the context and importance of TR4 in their home country. While it is true that we are not biologist or plant pathologists, we have shared the manuscript and the study was designed together with the expertise of a renowned plant pathologist.

**Reviewer #3**:

The article is not solid, mainly because information was obtained from farms that do not have Foc TR4, the data obtained from free farms makes the results hypothetical. The estimated costs are not adequate because they do not consider real costs of farms that implement biosecurity measures in the presence of the pathogen. The population sampled between both departments is very unbalanced and the analysis for the department of Antioquia and its distribution of types of producers is not representative.

Thank you very much for your comments. We have replied to each of your comments, with line numbers referring to those in the version with tracked changes.

We really appreciate the concerns you raised. While it is true that we did not include farms already affected by TR4 because they declined to participate in the study, the producers surveyed are actively implementing biosecurity measures specifically to prevent the entry of TR4. This means that the costs and benefits we analyzed are based on actual, ongoing practices, not hypothetical scenarios. We have also highlighted the fact that some of the data was provided by experts with several years of experience in the banana industry. This information was used to corroborate the information provided by the producers. The results were also shared with banana value chain actors, stakeholders, and subject matter experts at the national and regional level who also validated our results.

We agree that the sample is not balanced in the study areas; however, this was intentional due to the makeup of producers in the two distinct study areas. The reason for this is that there are mainly smallholder producers in Magdalena, whereas there are large holders and managers of large farms in Antioquia. Because of this, we were unable to increase the sample size in Antioquia. The two study regions were chosen for very specific reasons: they are the top two banana producing departments in Colombia; they have distinct farming systems so we could compare farming systems; they have different banana markets (export vs. domestic bananas); they mostly plant with Cavendish cultivators to produce export bananas; and one department (Magdalena) had three confirmed cases of TR4 when the study took place (lines 177-179). At the time of data collection, we were not able to conduct the study in other regions of Colombia or in other countries because of funding constraints. We had initially set out to conduct interviews in another department, La Guajira, which had confirmed cases of TR4 on large banana plantations; however, managers of these large plantations were unwilling to speak with enumerators, the authors, and hired consultants, let alone be interviewed, so we had to withdraw La Guajira from our study area. Despite the unbalanced data, we are confident that our data allows us to analyze the costs and benefits of implementing TR4 prevention measures. In addition, we took care to analyze the results by department, so that we did not mix producers from the two distinct study areas. The results and the table in the supplementary material show that there are statistically significant differences between the two study areas.

The title of the article has no relationship with the results and conclusions. There is a marked decontextualization of the Foc TR4 problem in Colombia and the productive realities of export bananas in that country. Socioeconomic impact indicators such as job losses are not considered in this study and that is significant for these regions that make a living from banana production.

Thank you for this comment. Based on this comment and that of another reviewer, we have revised our title to “A socioeconomic and cost benefit analysis of Tropical Race 4 (TR4) prevention methods among banana producers in Colombia.” Also, thank you for highlighting the importance of job losses in the Colombian banana sector. We have refined our manuscript by including an estimate of the number and value of indirect and direct job losses based on different spread rates of TR4 (see Table 5, line 735).

The article is not robust and does not represent new knowledge, due to the hypothetical nature of the results and its poor capacity for analysis.

Thank you for this feedback. We appreciate your honesty and criticism of our manuscript; however, our view is different. For example, our data is sourced from a variety of respondents and sources, both primary and secondary. In addition, we were able to determine several important insights into how to prevent the spread of TR4 through education and awareness. For example, common agricultural practices identified in this study that highlight the vulnerability of the area to the spread of TR4 include involve the use of corms from existing plots for replanting or establishing new plantations (lines 864-866). In addition, due to the comments from you and the other reviewers, we have improved the analysis and interpretation of the results. We also traveled to the field, visited farms, participated in workshops with banana value chain actors, and engaged with stakeholders and subject matter experts both before and after data collection (to validate our results) at the national and regional level. We also shared the manuscript and the study was designed together with the expertise of a renowned plant pathologist.

**Reviewer #4**:

General comments:

The paper by Ritter et al is an important assessment of the impact of TR4 in commercial and small-scale farming systems in Colombia. The authors could only work with the situation available, which includes the limited spread of the disease in the study areas, and the collaboration of growers. Still, the information obtained is important for the larger scientific community, especially the outcome that investment in preventative and mitigating measures can significantly increase the net value of banana production in both regions. The paper is reasonably well written, but the Discussion can be more comprehensive with more references.

First off, thank you for taking the time to read and provide such valuable feedback to our manuscript. We have replied to each of your comments, with line numbers referring to those in the version with tracked changes.

Thank you very much for this above recommendation. We have broadened the Discussion section by adding more references, such as: the impact of the disease on producers and the economy in other Latin American countries (line 775); trials of tolerant cultivators (lines 828-836); and other mitigation strategies identified and recommended in the literature for the management of TR4 (lines 841-864). We added a subsection in the Discussion section of the limitations of our study in which we refer to several other studies (lines 904-939). The subsection “Future research opportunities” (lines 940-967) also refers to more studies. Overall, throughout the revised manuscript, we have added significantly more references (about 24) per your suggestion and that of another reviewer.

I also have some specific questions and recommendations, which are listed below.

Title:

Why does the title of the manuscript on page 1 differ from that on page 3?

Thank you for noticing this discrepancy. The titles now match. In addition, we have slightly changed our title, which is now “A socioeconomic and cost benefit analysis of Tropical Race 4 (TR4) prevention methods among banana producers in Colombia.”

Abstract:

Page 3, line 46: ‘Susceptibility’ should rather be replaced with ‘vulnerability’. Also add the region to this sentence, i.e. Colombia.

Thank you for these suggestions. We have made these changes in the Abstract.

Introduction:

Page 4, line 66: Replace ‘targets’ with ‘blocks’.

Done.

Page 4, line 70: Please indicate what you mean with ‘but also raises concerns for various other crops’.

Thank you for posing this question. We added that other crops are susceptible to Fusarium, namely tomato, sweet potato, legumes, and cucurbits (the gourd family) (lines 82-83).

Page 4, line 70: What is meant with ‘Foc exhibits diverse populations’?

We added that Foc’s pathogens exhibit diverse and shifting populations as a result of changes in environmental conditions, as well as a citation of a study (Valverde-Bogantes et al., 2020) exploring this topic (line 84).

Page 4, line 70: In the same sentence it is stated that ‘TR4 being the sole strain capable of affecting the economically vital Cavendish cultivar’, which is not entirely true. Fusarium subtropical race 4 can also affect Cavendish bananas, although this happens in the subtropics. Also, do the authors consider Foc VCG 0121 a member of Foc TR4, as this strain can also cause disease to Cavendish bananas.

Thank you for pointing this out. We added that subtropical race 4 (STR4) can also affect Cavendish under certain abiotic constraints like water logging in sub-tropical regions, as you mentioned (García-Bastidas et al., 2022; Roberts et al., 2024) (lines 85-87). Along this same line, we also added that TR4 affects Cavendish and a wide range of local cultivators that are unaffected by R1 and R2 (Ploetz, 2015) (lines 89-90). In the first sentence of the paper, we now add that we consider VCG 0121/16 as a member of Foc TR4 (line 73).

Page 4, line 75: There is no proof that Foc TR4 is more aggressive than other races. It only affects more banana cultivars than the other races.

Thank you for pointing this out. We have revised the sentence and removed that it is the most aggressive strand (line 92).

Pg 4, line 79: TR4 was not identified in Asia in the 1970s. That is simply speculation. The fungus was only identified after affecting Cavendish bananas in Malaysia and Indonesia in the 1990’s.

Thank you for this historical detail. We have revised the sentence accordingly.

Pg 4, line 80: Please provide references for the occurrence of Foc TR4 in Africa, Colombia, Peru and Venezuela.

Thank you for this suggestion. We added references to the first confirmed occurrences of TR4 in each respective place.

Pg 4, line 88: Please provide a reference for the interventions implemented in Colombia.

We added the reference to this sentence.

Pg 5, lines 92-93: Please see de Figueiredo Silva et al. (2023). Estimating worldwide benefits from improved bananas resistant to Fusarium Wilt Tropical race 4. Journal of the Agricultural and Applied Economics Association 2(1).

Thank you for pointing out this study to us, which examines economic welfare implications of TR4 on global banana production rather than the effects within Latin America as a region or Colombia specifically. For this reason, we are still confident in our statement on these lines. However, we have now cited Figuiredo Silva et al. (2023) on line 128 in relation to the global consequences of TR4 and the delay in adopting resistant varieties.

Materials and Methods:

Page 6, lines 130-131: The sentence starting with ‘This section ….’ is unnecessary. Please remove.

Thank you. We have removed this sentence.

Page 6, lines 133-134. Rewrote this as follows: The departments of Antioquia and Magdalene in Colombia were selected due to several strategic considerations (Fig. 1).

Thank you. We have rewritten this sentence.

Page 6, line 134. Please start a sentence with a capital letter. It would also be acceptible if you start the sentence with ‘Firstly, these departments ….’.

Thank you for finding this typo. We have fixed it.

Page 7, line 157: Mention the name of your organization here.

Thank you. We added this to the sentence. We originally did not want to include it in the initial submission, so that the author’s organization would not be as obvious.

Page 8, line 173: ‘….as explained below.’

Corrected.

Page 10, line 244: Please correct this sentence by deleting one ‘steps’.

Corrected.

Page 12, line 260: This sentence starting with ‘This section …’ is unnecessary, and can be deleted.

Thank you. We have deleted this.

Page 15, line 329: Should the mean yield in Antioquia not be 46.24 instead of 44.1 tons/ha?

Thank you. We accidentally mixed mean and median from the two different departments in the same sentence. This is now fixed and we also specified that we are referring to the mean yield.

Page 16, line 341: The word ‘conditions’ can possibly be replaced with ‘differences’.

Thank you. We have adopted your suggestion.

Page 16, line 353: Fix $688 USD.

We did not change this as we have been using this format throughout the paper when referring to dollar values. We think it is important to clearly state that we are referring to USD when we use the $ symbol. We use the format $(value) USD throughout the paper.

Page 17, line 369: In section 3.2., more reference should be made to Table A1.

Thank you. We have added more references to Table A in S1 (formerly, Table A1).

Page 19, line 414: In section 3.2.2., please briefly describe the nature of the cement paths and fences. Are the paths between or in plantations, between blocks? Are the fences on farm borders, or within farms?

Thank you for these questions on the cement paths. The cement paths are delimited paths between the entrance to the farm, the place of production, and the administrative areas. Their purpose is to help minimize soil-to-plant contact, thereby reducing the risk of disease transmission. We have added this short explanation about cement paths at the beginning of the subsection “Cement paths and fences” (lines 533-543).

Page 21, lines 459-464: Not necessary to repeat this information that was already mentioned in the Methodology section.

Thank you. We have removed these lines. We thought they would be helpful for the readers, but in an effort to avoid redundancy, we deleted them.

Page 21, line 471: Table 3 provides a comprehensive overview ….

Corrected.

Pages 21, 22 and 23, sections 3.3.1., 3.3.2. and 3.3.3: Too much Methodology included in the beginning of these sections. Rather move the methods used to the Methodology sections.

Thank you for this recommendation. We have moved the methodology that was included in various parts of the results section into the Methodology (mainly incorporated into the steps of the CBA), as well as removed redundancies where necessary.

Page 23, line 511: This must be a mistake, as I cannot see the financial performance of biosecurity strategies being presented in Table 1. Should it be Table 5?

Yes, thank you. Some of our cross references/links to tables got their wires crossed when we converted it from a shared document online to an offline document to upload. We have fixed this.

Page 24, line 531: Refer to Table 5 in this section.

We now refer to this table (Table 4 in the revised version).

Page 25, line 551. Refer to Table 6 here.

Thank you. We now refer to this table (Table 5 in the revised version).

Discussion:

Page 27, lines 575-578: The first two sentences in this paragraph says essentially the same thing. Please merge or delete one of the sentences.

Thank you. We have merged these two sentences.

Page 27, line 578: The statement starting with ‘TR4 poses a serious threat ….’ is redundant and should rather be used in the Introduction.

Thank you. We have removed it from the Discussion section and incorporated it into the introduction.

Page 27, line 585: Please add references to this paragraph.

Thank you. We added references to this paragraph, namely Ploetz et al., (2015) and Thangavelu et al. (2019).

Page 27, lines 586-588: The authors should vindicate the statement ‘banana farms …. in both Antioquia and Magdalena are highly susceptible to the spread of TR4’ based on the findings of your study.

Thank you. We have added justification to this statement later in the paragraph in which we write: Common agricultural practices identified in this study that highlight the vulnerability of the area to the spread of TR4 include involve the use of corms from existing plots for replanting or establishing new plantations.

Page 27, line 588: Infested soil, not infected soil.

Corrected.

Page 28, lines 594-605: This paragraph is full of unsupported statements, such as that TR4 spread would lead to massive job losses, unemployment, and hunger. Why can these farms not grow other crops, for instance, to generate income? Are there any literature or experiences substantiating these statements? Maybe cite some work done in the early 1900s in Latin America following the Gros Michel – race 1 epidemic.

Thank you for these points of inquiry and suggestions. The literature acknowledges the role of humans in the spread of TR4 (Dita et al., 2018), which we have added to the manuscript. Dita et al. (2018) discuss the current spread and argue that the intercontinental movement of the pathogen suggests an influence of anthropogenic factors through the introduction of planting material, boots, or tools contaminated with Foc-contaminated soil to fungus-free areas. Furthermore, Dita et al. (2018) comment that the case is similar to the spread of Foc R1, where the dispersal of asymptomatic infected material was a fundamental factor in the devastation of Gros Michel bananas in the 1900s, which we now include (lines 841-846).

Colombia has regulations on the use of TR4 contaminated land: Subject matter experts and stakeholders stated in various workshops and meetings that growing bananas or any other crops on land infected by TR4 is not permitted, and that if producers want to do anything on such land, they need special permission by ICA (ICA, 2024), which authorizes activities to be carried out in quarantined areas. Among the recommendations, experts mention that productive activities should not be carried out in infected areas to reduce the risk of the fungus reaching other areas. Because of this, producers would not be able to grow other crops on land already infected by TR4. We have made this point more explicit in the paragraph you highlighted in your comment (which now starts on line 794). In addition, we have removed a lot of the unsupported statements (lines 797-808). Lastly, we specified that producers may already need to find more suitable crops to grow, such as paddy rice, sugarcane, tapioca, onion, and pineapple which are non-host crops for TR4 and reduce the inoculum level of the pathogen (Thangavelu and Uma, 2019), or off-farm employment opportunities to help hedge the risk of TR4 spreading on their farms (Montenegro-Gracia et al., 2021) (lines 808-812).

Page 28, line 606: This section also requires some references for certain statements, especially on pro-active measures as the main means by which TR4 can be mitigated, and on the availability of resistant varieties. Please add these.

Thank you, we have added information on tolerant cultivators, along with references (lines 828-836). For example, we state that trials of tolerant cultivars – including FHIA hybrids and Cavendish clones like GCTCV-218 and GCTCV-119 – are being carried out to protect future banana crops and ensure sustainable production in the region (Blomme et al., 2024; Mintoff et al., 2021; Rocha et al., 2021). In addition, although Cavendish somaclones, like 'Formosana' (GCTCV-218) demonstrate intermediate TR4 tolerance, they may not meet the stringent quality standards of international markets (Blomme et al., 2024). We added a paragraph that discusses other strategies identified and recommended in the literature for the management of TR4 (lines 841-864). Here, we mention prevention protocols established by ICA, as well as those mentioned in the literature.

Page 29, line 614: It is not true that there are no banana varieties resistant or tolerant to TR4. Both FHIA-1 and GCTCV-218 (Formosana) are resistant and tolerant, respectively. Consult the literature on this. There might be other reasons why these are not acceptable for planting in Colombia.

Thank you for this comment. On lines 830-836, we added a clarification on resistant varieties, including FHIA hybrids. In addition, we write that despite significant research efforts to identify TR4 resistant banana cultivars, commercially available dessert bananas with agronomic traits comparable to Cavendish and matching Cavendish's tolerance to Foc Race 1 remain elusive. Bred hybrids, such as the FHIA lines, often exhibit non-ideal fruit characteristics or lower yields, hindering their large-scale adoption for export (Blomme et al., 2024). Similarly, although Cavendish somaclones, like 'Formosana' (GCTCV-218) demonstrate intermediate TR4 tolerance, they may not meet the stringent quality standards of international markets (Blomme et al., 2024).

Page 29, lines 615-617: What do these statistical regional differences mean? This should also be discussed here, and not only the reason for the differences.

Thank you for pointing out this confusion. We have made this clearer in the revised version. We were referring to Table A in S1 (formerly, Table A1), which finds that there are statistically significant differences in the adoption of some measures when comparing farmers in Antioquia and Magdalena.

Page 30, line 642: I appreciate that the current study is very unique, and based on predictions. If possible, however, one hope that the authors could compare their results to what happened in terms of the financial and social impact during the Gros Michel-Foc race 1 epidemic in Latin America in the early 1900s.

Thank you for this comment. We have added a comparison based on Dita et al. (2018) to the Gros Michel cultivator of bananas in the 1900s (lines 843-845).

Page 31, line 666 onwards: There are also other interventions that need to be mentioned here. For instance, the fencing of properties and regulation of access, the digging of trenches and canals to direct the flow of water. Disinfection is a science by its own, and should be correctly used, while cement paths are not affordable by all.

Thank you for this suggestion. While we are aware that there are other interventions, they were not mentioned by the surveyed producers. For example, although there are recommendations from ICA on how to reduce the spread of TR4 (such as good crop nutrition, maintenance of drainage networks and water conduction channels, and avoiding the dispersal of plant material in water sources and drainage channels), these practices were not mentioned by the surveyed producers. This shows that increased awareness of these other mitigation measures is needed. We have added a paragraph in the limitations subsection that includes these other interventions (lines 916-926).

Page 31, line 679-683: The authors also discuss what government, cooperatives and associations should do, but not what they have already done, or not done. That would provide some perspective.

Thank you, we have added that the Colombian government together with cooperatives agreed to invest nearly $7.2 million USD in TR4 prevention, surveillance, and awareness measures from 2019-2023 (lines 994-998). Unfortunately, there is no data on what is planned to be invested in the future. We have also made it clearer at the end of this paragraph that support for producers is also needed to help them obtain agricultural inputs to help relieve their financial constraints (lines 999-1001).

Pg 32, line 704: The use of drones to detect TR4 is not well proved and should be recommended with care. Rather explain that it should be used to identify symptomatic or suspect plants that must be further investigated.

Thank you for this helpful suggestion. We have added the following sentence to this paragraph (on lines 1022-1024): Drones could be used to identify symptomatic or suspect plants as well as areas prone TR4 that deserve further investigation through other techniques (Tagami et al., 2024).

**References**

Alvarez D, Mosquera L, Ritter T, Mockshell J, Dita,M. Banana value chain profile for Colombia: Production and Fusarium Tropical Race 4 (TR4) at a crossroad. Cali (Colombia): Bioversity International and International Center for Tropical Agriculture (CIAT). 2023.10 p.

Blomme G, Kearsley E, Ocimati, W, Dita, M. Fusarium Wilt of Banana: Training manual. Version 1. Bioversity International, Rome, Italy, 2024.

Dita M, Barquero M, Heck D, Mizubuti ESG, Staver CP. Fusarium Wilt of Banana: Current Knowledge on Epidemiology and Research Needs Toward Sustainable Disease Management. Front. Plant Sci. 2018. doi: 10.3389/fpls.2018.01468.

García-Bastidas FA, Arango-Isaza R, Rodriguez-Cabal HA, Seidl MF, Cappadona G, Segura R, et al. Induced resistance to Fusarium wilt of banana caused by Tropical Race 4 in Cavendish cv Grand Naine bananas after challenging with avirulent Fusarium spp. PLoS ONE. 2022;17(9). doi: 10.1371/journal.pone.0273335.

Instituto Colombiano Agropecuario (ICA). Resolución ICA 2081, March 11, 2024. Bogotá, Colombia.

Mintoff SJL, Nguyen TV, Kelly C, Cullen S, Hearnden M, Williams R, et al. Banana Cultivar Field Screening for Resistance to Fusarium oxysporum f.sp. cubense Tropical Race 4 in the Northern Territory. J Fungi (Basel). 2021;7(8). doi: 10.3390/jof7080627.

Montenegro-Gracia EJ, Pitti-Rodríguez JE, Olivares-Campos BO. Identification of the main subsistence crops of Teribe: a case study based on multivariate techniques. Idesia (Arica). 2021;39(3):83-94. doi:10.4067/S0718-34292021000300083

Ploetz RC. Fusarium wilt of banana. Phytopathology. 2015;105(12): 1512-1521. doi: 10.1094/PHYTO-04-15-0101-RVW.

Ploetz R, Freeman S, Konkol J, Al-Abed A, Naser Z, Shalan K, et al. Tropical race 4 of Panama disease in the Middle East. Phytoparasitica. 2015;43: 283-293. doi: 10.1007/s12600-015-0470-5.

Roberts JM, Carvalhais LC, O'Dwyer C, Rincón‐Flórez VA, Drenth A. Diagnostics of Fusarium wilt in banana: Current status and challenges. Plant Pathol. 2024 Jan 22;73(4):760–76 doi: 10.1111/ppa.13863.

Rocha A, Soares J, Nascimento F, Santos A, Amorim, V, Ferreira C, et al. Improvements in the Resistance of the Banana Species to Fusarium Wilt: A Systematic Review of Methods and Perspectives. J. Fungi. 2021; 7:249. doi: 10.3390/jof7040249.

Silva F, Kaplan S, Magdama F, Potts M, Martinez R, Zilberman D. Estimating worldwide benefits from improved bananas resistant to Fusarium Wilt Tropical race 4. Journal of the Agricultural and Applied Economics Association. 2023 Jan 19;2. doi:10.1002/jaa2.41.

Tagami GN, van Westerhoven AC, Seidl MF, van der Sluis J, Cozzarelli M, Balarezo Camminati D et al. Aerial mapping of the Peruvian Chira Valley banana production system to monitor the expansion of Fusarium wilt caused by Tropical Race 4. PhytoFront. 2024 Apr 16:PHYTOFR-07. doi: 10.1094/PHYTOFR-07-23-0097-R.

Thangavelu R, Mostert D, Gopi M, Devi PG, Padmanaban B, Molina AB, et al. First detection of Fusarium oxysporum f. sp. cubense tropical race 4 (TR4) on Cavendish banana in India. Eur. J. Plant Pathol. 2019 Jul 15;154: 777-786. doi: 10.1007/s10658-019-01701-6.

Valverde-Bogantes E, Bianchini A, Herr JR, Rose DJ, Wegulo SN, Hallen-Adams HE. Recent population changes of Fusarium head blight pathogens: drivers and implications. Canadian Journal of Plant Pathology. 2020 Jul 2;42(3):315–29. doi: 10.1080/07060661.2019.1680442.
